# Pathways for Potential Exposure to Onshore Oil and Gas Wastewater: What We Need to Know to Protect Human Health

**DOI:** 10.1029/2024GH001263

**Published:** 2025-04-03

**Authors:** Ayusha Ariana, Isabelle Cozzarelli, Cloelle Danforth, Bonnie McDevitt, Anna Rosofsky, Donna Vorhees

**Affiliations:** ^1^ Health Effects Institute Energy Boston MA USA; ^2^ Geology, Energy & Minerals Science Center U.S. Geological Survey Reston VA USA

**Keywords:** produced water, human exposure, human health, oil and gas wastewater, water reuse

## Abstract

Produced water is a chemically complex waste stream generated during oil and gas development. Roughly four trillion liters were generated onshore in the United States in 2021 (ALL Consulting, 2022, https://www.gwpc.org/wp‐content/uploads/2021/09/2021_Produced_Water_Volumes.pdf). Efforts are underway to expand historic uses of produced water to offset freshwater needs in water‐stressed regions, avoid induced seismic activity associated with its disposal, and extract commodities. Understanding the potential exposures from current and proposed produced water uses and management practices can help to inform health‐protective practices. This review summarizes what is known about potential human exposure to produced water from onshore oil and gas development in the United States. We synthesize 236 publications to create a conceptual model of potential human exposure that illustrates the current state of scientific inquiry and knowledge. Exposure to produced water can occur following its release to the environment through spills or leaks during its handling and management. Exposure can also arise from authorized releases, including permitted discharges to surface water, crop irrigation, and road treatment. Knowledge gaps include understanding the variable composition and toxicity of produced water released to the environment, the performance of treatment methods, migration pathways through the environment that can result in human exposure, and the significance of the exposures for human and ecosystem health. Reducing these uncertainties may help in realizing the benefits of produced water use while simultaneously protecting human health.

## Key Points

- Oil and gas wastewater (produced water) is a complex mixture with management challenges and opportunities
- Use of produced water outside the oil field can reduce freshwater demands and alleviate disposal hurdles
- Knowledge gaps remain regarding potential human exposure to produced water

## 1 Introduction

Oil and gas wastewater, known as produced water, is a combination of naturally occurring water from the subsurface environment and the water and chemicals injected into the well during its development or maintenance (“American Geosciences Institute,” 2016; Engle et al., 2014). Produced water returning to the surface soon after a well is completed is sometimes called flowback. The proportion of produced water that consists of naturally occurring water, or formation water, increases over the life of a hydraulically fractured well (Rowan et al., 2015). In this review, we use produced water to refer to any liquid surfaced from an oil or natural gas well (Bean et al., 2018; Engle et al., 2014). Produced water can flow to the surface for months to years until the production of oil or gas ceases (Butkovskyi et al., 2017). In 2021, an estimated 4.2 trillion liters of produced water was generated onshore in the United States (Groundwater Protection Council, 2023).

**Figure 1:**
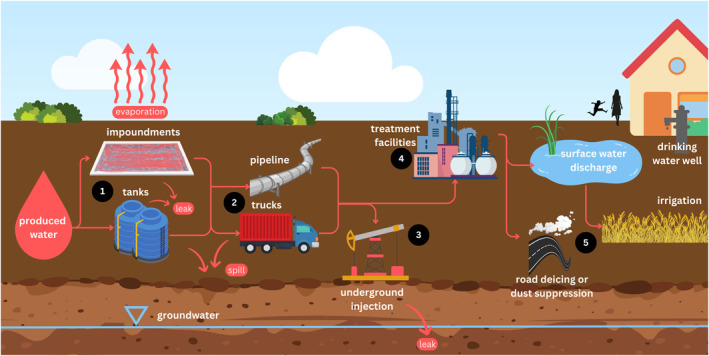
A general summary of produced‐water management options and potential routes of exposure. Management of produced water can include its (1) collection and storage, (2) transport, (3) disposal, primarily through Class II Underground Injection Control (UIC) wells, as represented by “underground injection,” (4) treatment, and (5) potential uses such as irrigation or road de‐icing or dust suppression (ALL Consulting, 2022; Groundwater Protection Council, 2023). During its management, produced water might be released to the environment through spills, leaks, or evaporation from open‐air storage impoundments or through authorized discharges.

Management of produced water can include its collection, storage, transport, treatment, and disposal (Figure 1). Disposal of produced water occurs mainly through Class II Underground Injection Control (UIC) wells, which are regulated under the Safe Drinking Water Act (U.S. Environmental Protection Agency, 2020). Produced water is sometimes recycled for use in developing oil and gas wells, discharged to surface water, or used outside of the oil field for purposes unrelated to oil and gas development (Groundwater Protection Council, 2023; U.S. Environmental Protection Agency, 2020). Most produced water, approximately 98%, is deep‐well injected for disposal or used in enhanced oil recovery or for subsequent hydraulic fracturing in the oil field (ALL Consulting, 2022). Produced water can require differing levels and types of treatment depending on its disposition or intended end use, and general management practices differ across regions due to variations in the composition and quantity of produced water, industry practices, and governance of oil and gas operations. Produced water also might be released to the environment through spills or leaks from storage or transport infrastructure, or evaporation from open‐air storage impoundments.

Produced water can be treated for uses unrelated to oil and gas operations such as crop irrigation, livestock watering, and road treatment to suppress dust and deice (Shariq, 2013; State of New Mexico & U.S. Environmental Protection Agency, 2018; Texas Produced Water Consortium, 2022; U.S. Environmental Protection Agency, 2020). Treatment methods continue to be developed to support various end uses (Cooper et al., 2022). In several water‐stressed regions of the United States, initiatives are underway to expand on historic uses of produced water outside the oil field to offset and reduce freshwater needs and avoid induced seismic activity associated with its disposal (Cooper et al., 2022; Sabie et al., 2022; Scanlon et al., 2020). Extraction of lithium from produced water is the subject of growing interest and inquiry as demand for critical minerals increases to produce batteries and other goods (Knierim et al., 2024; Kumar et al., 2019; Mackey et al., 2024; McDevitt et al., 2024; Smith et al., 2024).

Understanding knowledge gaps related to human exposure to produced water is important as U.S. states seek opportunities to use produced water outside the oil field. Both Texas and New Mexico are currently exploring beneficial use opportunities for produced water. The New Mexico Produced Water Research Consortium seeks to develop the science around produced‐water treatment and use to inform the New Mexico Environment Department (NMED) policies and rules on produced‐water use outside the oil field. The NMED drafted rules that specify how produced water may be used in pilot studies but explicitly prohibits the discharge of produced water to the environment before the rules are finalized by the Water Quality Control Commission (New Mexico Environment Department, 2024). The Texas Produced Water Consortium is pursuing similar pilot studies for produced water use outside the oil field. The Texas Railroad Commission released the *Produced Water Beneficial Reuse Framework for Pilot Study Authorization* that describes how to obtain authorization for treated produced‐water pilot studies for land application (Railroad Commission of Texas, 2024), and the Consortium is seeking applications to understand testing requirements needed for produced‐water discharge to surface waters (Texas Produced Water Consortium, 2024). Recently, the Texas Commission on Environmental Quality renewed a permit for the discharge of produced water into surface water (Texas Commission on Environmental Quality, 2024).

With pressure growing to expand on current uses of produced water outside the oil field, we aim to synthesize the current understanding of how people might be exposed to produced water and identify knowledge gaps to help inform policies to protect human health.

## 2 Methods

**Figure 2:**
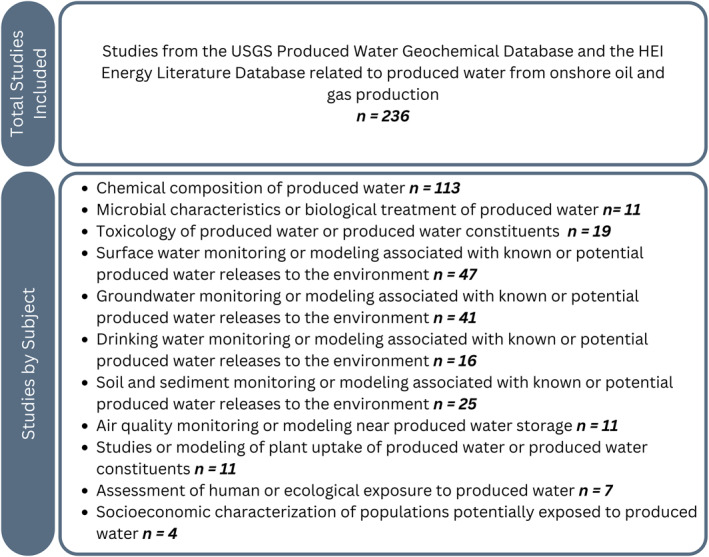
A summary of the publications discussed in this review. Many of the studies address more than one environmental medium (e.g., air, water, and soil) or type of analysis (e.g., toxicology and exposure assessment); therefore, the total number of “Studies by Subject” cannot be summed to the total number of studies in this review (*n* = 236).

In this study, we update and expand on a summary of 112 publications on potential human exposures to produced water from onshore oil and gas production in the United States (Ariana et al., 2023). This review expands on that summary for a total of 236 publications from the peer‐reviewed scientific literature and gray literature (Figure 2), discussion of additional topics (i.e., produced‐water toxicity, microbial characteristics), and includes a commentary on remaining knowledge gaps about potential human exposures to produced water.

Literature published between 1 January 2000, and 15 December 2023, was identified by searching the U.S. Geological Survey (USGS) Produced Water Geochemical Database (Blondes et al., 2019) and the HEI Energy Literature Database (Ariana et al., 2023), both of which are open source (HEI Energy Research Committee, 2020).For the HEI Energy Literature database, peer‐reviewed literature is identified using four electronic databases: PubMed (https://www.ncbi.nlm.nih.gov/pubmed/), Web of Science (https://www.webofknowledge.com/), Embase (https://www.embase.com/) and Google Scholar (https://scholar.google.com/) and the Boolean search phrases described in the survey of the UOGD exposure literature (HEI Energy Research Committee, 2020).

This review focuses on studies that (a) characterized produced water and its toxicity, (b) described or documented how chemicals found in produced water behave and migrate following release to the environment, (c) conducted human health risk assessments or exposure assessments, or (d) characterized socioeconomic characteristics of potentially exposed populations.

Studies about produced water from conventional oil and gas development (COGD) and UOGD are included, and we highlight publications that distinguish between the two types of development (Gregory et al., 2011; Kharaka et al., 2013). We exclude studies on produced water from coalbed methane development and from development occurring offshore or outside the United States. For studies including transnational basins, we review only the aspects located within the United States.

**Figure 3:**
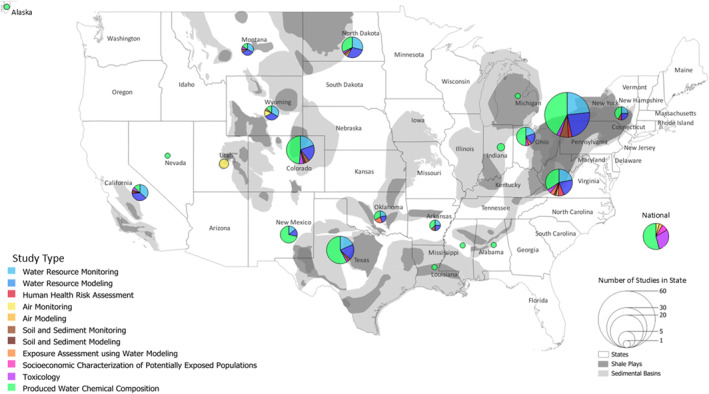
Map of shale plays and sedimentary basins in the United States (U.S. Energy Information Administration, 2019) and study locations by state included in this review represented by type of study. Hawai'i is not included due to the lack of unconventional oil and gas development (UOGD) in the state. Studies that collected or used data from multiple states are cross listed in the number of studies for each state, which is indicated by pie chart size. Additional studies that describe produced water on a national scale were not mapped but are included in Tables S1 and S2 in Supporting Information S1.

Figure 3 illustrates the distribution of studies across the United States organized by type of study, and Tables S1 and S2 in Supporting Information S1 summarize each study's location, data sources, exposure medium, and whether the produced water is from UOGD, COGD, or both. The highest number of studies took place in Pennsylvania (71), Texas (32), Colorado (32) and West Virginia (31). Most studies provided information on the chemical composition of produced water (113) and monitored chemical concentrations in water bodies adjacent to infrastructure associated with oil and gas development (77). Some studies measured the toxicity of produced water (18), measured chemicals in nonaqueous environmental media such as soil and sediment (17) and air (7), assessed exposure to produced water (7), and characterized populations potentially exposed to produced water (4).

## 3 Discussion

We frame discussion of the literature within a conceptual model of human exposure that illustrates exposure pathways potentially connecting produced water and people. The conceptual model does not necessarily include all possible exposure pathways; instead, it summarizes those investigated in the reviewed literature. The discussion begins with a description of produced water, the various exposure pathways assessed in the literature, and potentially exposed populations. We end with a discussion of gaps in understanding about potential exposures and recommendations for addressing them.

### 3.1 Produced Water Composition

#### 3.1.1 Geochemical Properties

Studies from several oil‐ and gas‐producing regions reported on the chemical composition of produced‐water quality (Figure 3 and Table S2 in Supporting Information S1). Produced water contains varying levels of salinity (approximately five to 10 times higher than seawater in some locations), total dissolved solids (TDS), dissolved organic matter, total suspended solids, metals, metalloids such as arsenic, critical minerals, volatile and semi‐volatile organic compounds (VOCs and SVOCs) including BTEX (benzene, toluene, ethylbenzene, and xylene) and PAHs (polycyclic aromatic hydrocarbons), naturally occurring radioactive material, ammonia, chemical additives such as surfactants and biocides, and per‐ and polyfluoroalkyl substances (Akob et al., 2015; Al‐Ghouti et al., 2019; Chen et al., 2023; Dresel & Rose, 2010; Gallegos et al., 2021; Jiang et al., 2022; Kumar et al., 2019; Lester et al., 2015; Liden et al., 2022; Luek & Gonsior, 2017; McDevitt et al., 2022; Murali Mohan, Hartsock, Hammack, et al., 2013, Murali Mohan, Hartsock, Hammack, et al., 2013; Orem et al., 2014; Rosenblum, Nelson, et al., 2017; Schreiber & Cozzarelli, 2021; Sun et al., 2019; Thakur et al., 2022; U.S. Geological Survey, 2022; Ziemkiewicz, 2013). In some instances, specific constituents in produced water reportedly exceeded U.S. Environmental Protection Agency (U.S. Environmental Protection Agency, 2020) drinking water standards (Chittick & Srebotnjak, 2017; Ziemkiewicz & He, 2015).

Certain properties, including ion ratios, isotopic compositions (e.g., ^87^Sr/^86^Sr), and the concentration and type of halogenated organic compounds, can serve as tracers to distinguish the influence of injected fluid versus formation water, produced water from different types of energy development (e.g., UOGD and COGD), and wells of varying ages (Barnaby et al., 2004; Cantlay et al., 2020; Goldberg & Griffith, 2017; Luek & Gonsior, 2017; Ogbuji et al., 2022; Osborn & McIntosh, 2010; Peterman et al., 2012; Phan et al., 2018; Sirivedhin & Dallbauman, 2004; Warner et al., 2014). Elevated concentrations of sodium, chloride, barium, strontium, lithium, strontium and radium isotopic compositions, and trace hydrocarbons were determined to be key markers of a large pipeline spill of produced water in North Dakota (Cozzarelli et al., 2017, 2021; Lauer et al., 2016). Strontium (Sr) isotopic compositions (^87^Sr/^86^Sr) can provide robust tracers of salinity sources, such as produced water, through the environment due to their conservative environmental behavior (Chapman et al., 2012; Cozzarelli et al., 2016; Harkness et al., 2017; McDevitt, McLaughlin, et al., 2020; Warner et al., 2014). Studies have indicated the ability to utilize ^87^Sr/^86^Sr to identify and quantify the accumulation of produced‐water contaminants in both mussel shells and tissues even with the application of low doses of produced‐water exposure (i.e., 0.15%) (Geeza et al., 2018; McDevitt, Geeza, et al., 2021). These data can be used to differentiate sources of contaminants that originate from spills and leaks of produced water from legacy contaminants from other oil and gas and industrial activities. This source apportionment is essential to evaluating the relative contribution from multiple potential routes of exposures. Thus, isotopic tracers such as ^87^Sr/^86^Sr can provide a unique identifier of potential produced‐water exposure pathways to humans.

The complex composition of produced water creates unique challenges to measuring chemical components, treatability for potential use, and understanding effects once released to the environment. The high salinity of produced water can make chemical analysis and treatment difficult. For example, salinity can be an obstacle to the accurate measurement of organic and inorganic constituents because methods are typically designed and optimized for surface water or groundwater that has relatively lower salinity. Consequently, even where produced‐water chemical characterization data exist, it may be incomplete or even unreliable, driving the need for method development to overcome these challenges (Jubb et al., 2020; Oetjen et al., 2017; Santos et al., 2019; Tasker et al., 2019). Furthermore, produced water's high salinity levels can foul treatment membrane systems and can be toxic to microorganisms commonly used in biological treatment systems (Acharya et al., 2020; Akyon et al., 2018). Constituents in produced water can affect the rate of biodegradation necessary to remove organic material (Akyon et al., 2018; Tinker et al., 2020, 2022). Studies recommend various approaches to address these challenges, including hybrid treatment approaches to address the complex assemblage of constituents (Acharya et al., 2020; Akyon et al., 2018; Hildenbrand et al., 2018; Tinker et al., 2020, 2022) and co‐treatment of abandoned mine drainage and produced water to manage radioactivity (He et al., 2016; McDevitt, Cavazza, et al., 2020; Ouyang et al., 2019).

#### 3.1.2 Variability in Composition

**Figure 4:**
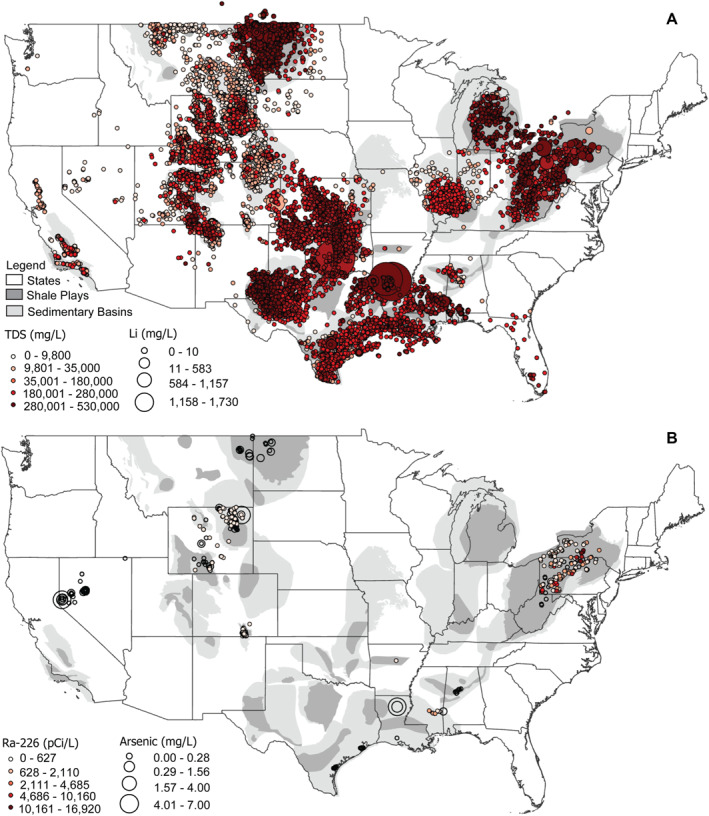
Map of the United States showing concentration distributions for (a) total dissolved solids (TDS) and lithium (Li) (*n* = 7,280) and (b) arsenic (*n* = 539) and radium‐226 (*n* = 727) in produced water. The data are from the U.S. Geological Survey National Produced Waters Geochemical Database (Blondes et al., 2019). Efforts are underway to extract Li from produced water given the growing demand for this mineral to produce energy storage batteries and other goods (Knierim et al., 2024; Kumar et al., 2019; Mackey et al., 2024; McDevitt et al., 2024). Note that while ample TDS concentration data exist, data are sparse for many constituents of potential concern for health, such as arsenic and radium‐226. Identifying geospatial data gaps illustrated by these produced‐water composition maps is important to understand the potential significance of any human exposures that might arise from produced‐water uses.

The composition of produced water varies as a function of the geologic formations from which the oil or gas is sourced, the composition of the oil or gas resource, the amount of time since production began, the type of drilling, the composition and volume of hydraulic fracturing fluid used to complete the well, and well maintenance procedures (U.S. Environmental Protection Agency, 2020). For example, Figure 4a demonstrates the wide variability of produced‐water composition by displaying concentrations of TDS, which can range over six orders of magnitude across the U.S., and lithium across shale plays and geological basins (Blondes et al., 2019; Nicot et al., 2018; Wang, Lu, et al., 2019). Figure 4b shows known carcinogens radium‐226 (Ra‐226) (*n* = 727 ranging from non‐detect to 16,920 pCi/L) and arsenic (*n* = 539 ranging from non‐detect to 7 mg/L) concentrations in contiguous U.S. produced water. Highest Ra‐226 concentrations measured in produced‐water samples were collected from the Appalachian Basin while highest arsenic concentrations were collected from the Arkla Basin. Figure 4b additionally provides visual perspective on the sparsity of select trace element data important for understanding human exposure compared to the plethora of produced‐water sample data available for more commonly measured inorganic salt species such as TDS.

Produced‐water composition can also vary between samples retrieved from a single well, shale play, or basin (Abualfaraj et al., 2014; Akob et al., 2015; Blauch et al., 2009; Chaudhary et al., 2019; Goldberg & Griffith, 2017; Jiang et al., 2021; Kim et al., 2016; Macpherson, 2015; McMahon et al., 2018; Rowan et al., 2015; Varonka et al., 2020; Welch et al., 2021). Produced‐water sampling often occurs at a variety of collection points besides a specific wellhead or separator, and so can include an aggregated mixture of produced water from multiple wells and/or geologic units such as storage tanks situated on well pads (Mackey et al., 2024) or prior to injection into saltwater disposal wells (Jiang et al., 2022). Concentrations of several constituents can also vary as the ratio of formation water and injected fluid increases over the lifespan of a well (Hayes, 2009; Oetjen et al., 2018; Oetjen & Thomas, 2016). Part of this variability comes from the different hydraulic fracturing fluid additives used for the specific environmental conditions of a given well and how those additives react in the subsurface environment (Kim et al., 2019; Ziemkiewicz & He, 2015). Investigations continue to identify hydraulic fracturing fluid additives in produced water and whether they can be used as tracers in investigations of produced water as potential contamination sources (Lester et al., 2015; Nell & Helbling, 2019; Rosenblum, Thurman, et al., 2017; Sitterley et al., 2018; Thacker et al., 2015; Thurman et al., 2017).

#### 3.1.3 Microbiological Characteristics

Microbial activity in produced water can cause corrosion of well equipment and potentially lead to environmental releases (Booker et al., 2017; Chilkoor et al., 2018; Daly et al., 2016; Liang et al., 2016; Lipus et al., 2017; Vikram et al., 2016). Microbial composition of produced water changes over time (Lipus et al., 2018) and can resist the efficacy of biocidal and disinfection processes, which can compromise well casing and grout integrity (Cluff et al., 2014; Hildenbrand et al., 2018; Struchtemeyer & Elshahed, 2012). Under thermophilic and anaerobic conditions, microbes can also metabolize hydrocarbons (Gieg et al., 2010) and polysaccharides in hydraulic fracturing fluid additives (Liang et al., 2016; Wang, Lu, et al., 2019). Pretreatment to mitigate solid formation and microbial activity before storage can help prevent corrosion and scaling (Murali Mohan, Hartsock, Bibby, et al., 2013; Thiel & Lienhard V, 2014).

#### 3.1.4 Produced Water Toxicology

A subset of the literature focused on toxicological effects of produced water, including original research and reviews about the toxicity of chemicals associated with produced water. Several studies looked at the toxicity of produced water to aquatic organisms and systems. Larval amphibian tissue contained concentrations of selenium, vanadium, and other ions associated with contamination from produced water in the wetlands of Montana and North Dakota (Hossack et al., 2018; Preston et al., 2019; Smalling et al., 2019). The study of a contamination event from a pipeline spill in North Dakota used a biological multi‐level investigation approach and found increased mortality in fish and elevated estrogenic activity initially (6 months post‐spill), which decreased over time (3 years post‐spill), while changes in microbial community structure persisted (Farag et al., 2022). Based on liver tissue samples from Brook trout in watersheds with active natural gas development in the Marcellus region in Pennsylvania, McLimans et al. reported potential disruptions to endocrine activity and indications of stress responses (McLimans et al., 2022). Other studies reported accumulation of produced‐water constituents in the shell and soft tissue of freshwater mussels (Geeza et al., 2018; McDevitt, Geeza, et al., 2021; Patnode et al., 2015; Piotrowski et al., 2020; Wang, Kunz, et al., 2019). Wang et al. reported decreased survival and growth rates in both fish and mussels and McDevitt et al. reported accumulation of produced‐water constituents in freshwater mussels even at low doses of exposure to produced water, which could make mussels a good indicator of produced‐water contamination (McDevitt, Geeza, et al., 2021; Wang, Kunz, et al., 2019). However, investigators generally suggested conducting larger studies with greater replicability to confirm these results (Geeza et al., 2018; Patnode et al., 2015; Piotrowski et al., 2020).

A suite of studies examined health outcomes from exposing adult and pregnant mice during preconception, prenatal, postnatal, and lactational phases to (a) a mixture of 23 commonly used hydraulic fracturing fluid additives, (b) samples of UOGD produced waters from Colorado and West Virginia, and (c) related samples of surface and ground waters impacted by produced water in those regions (Balise, Cornelius‐Green, Parmenter, et al., 2019, Balise, Cornelius‐Green, Parmenter, et al., 2019; Boulé et al., 2018; Kassotis et al., 2015, 2016, 2018b; Nagel et al., 2020; Orem et al., 2017). They reported effects on hormone concentrations, adipogenic (fat cell‐related) activity, energy expenditure, and risk‐taking behavior in mice. They also reported associated effects such as decreased sperm counts, increased body weight, altered uterine and ovary weights, increased heart weights and collagen deposition, disrupted folliculogenesis, increased mammary gland ductal density, and increased preneoplastic lesions, representing adverse developmental and reproductive health outcomes in wildlife when exposed to this chemical mixture. Exposing human tissue culture cells to the same chemical mixture, studies reported impacts on estrogen, androgen, glucocorticoid, progesterone, and thyroid receptor activity, representing potential endocrine disruptions (Kassotis et al., 2014, 2018a; Nagel et al., 2020). Organic chemicals and additives were specifically associated with higher acute toxicity (Aghababaei et al., 2021) and tumor promotion (Crosby et al., 2018). Yao et al. reported that exposure to flowback from the Marcellus Shale can induce malignant cell transformation in vitro, suggesting carcinogenic effects (Yao et al., 2015).

Compiling data and knowledge on produced‐water toxicity to different species under varying environmental conditions allows for more refined risk and hazard assessments that clarifies possible effects to human health if exposed (Danforth et al., 2019; Elliott et al., 2016; Rose et al., 2019). Some investigators have recommended more study to assess the combined toxicity of produced‐water constituents (Crosby et al., 2018) and chronic toxicity resulting from National Pollutant Discharge Elimination System (NPDES)‐permitted discharges and the extent of exposure downstream (McDevitt, Geeza, et al., 2021; McLaughlin, Blotevogel, et al., 2020, McLaughlin, Borch, et al., 2020).

### 3.2 Potential Exposure Pathways Examined in the Literature

**Figure 5:**
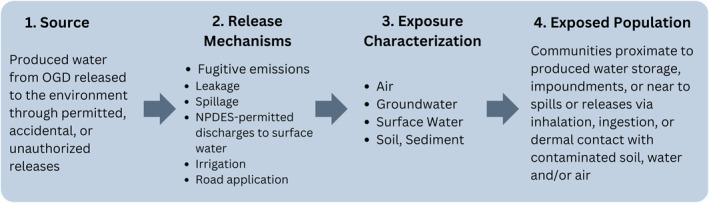
Simplified conceptual model of potential exposure pathways associated with oil and gas development (OGD) produced‐water management from onshore oil and gas development. The pathways reflect the literature summarized in this brief and do not necessarily include all possible exposure pathways. NPDES = National Pollutant Discharge Elimination System. Figure reproduced with permission from Ariana et al. (2023).

The discussion of this literature is organized in accordance with a conceptual model of potential exposure pathways assessed in the literature (Figure 5). We begin with produced water released to the environment through a variety of permitted, fugitive, and accidental releases, and follow it through potentially contaminated environmental media, and to the human populations that may be exposed. Because the model is limited to what was assessed in the literature, it might not depict all possible produced‐water exposure pathways.

#### 3.2.1 Accidental or Fugitive Release of Produced Water to the Environment

Evaporation, spills, or leaks from well equipment such as onsite storage tanks, spills during produced water transportation, and accidental discharges to surface water represent unintentional or intentional produced‐water releases to the environment (Abraham et al., 2023; Akob et al., 2016; Bain et al., 2021; Bean et al., 2018; Boulé et al., 2018; Drollette et al., 2015, 2015, 2015; Kashani et al., 2024; Kassotis et al., 2016; Llewellyn et al., 2015; Maloney et al., 2017; McMahon et al., 2017; Orem et al., 2017; Reilly et al., 2015; Rish & Pfau, 2018; Wang, 2021; Warner, Christie, et al., 2013; Wright et al., 2019). These releases may impact ecosystem health and could also result in human exposures to produced‐water constituents in air, soil, sediment, surface water, and groundwater.

***Emissions to Air***. Emissions from open‐top produced‐water storage impoundments can contribute to particulate matter (PM), VOCs, and other constituents of concern in ambient air (Bean et al., 2018; Ma et al., 2022). Repeated recycling of produced water from hydraulic fracturing concentrates constituents like radium in the “impoundment sludge” that accumulates at the bottom of storage tanks, which then subsequently radioactively decays and can be released into the air as carcinogenic radon gas. Zhang et al. investigated the potential for radiation exposure from impoundment sludge in the Marcellus (Zhang et al., 2015). To avoid risks to humans from unintentional radiation exposure, the researchers proposed management options such as transporting impoundment sludge to low‐level radioactive waste landfills, hazardous waste landfills, or municipal and industrial solid waste landfills (Zhang et al., 2015). In their exposure assessment, the authors further reported low carcinogenic risks associated with inhaling radon emissions from this sludge for on‐site workers and the surrounding public (Zhang et al., 2015).

Studies have also reported on fugitive emissions from produced water at the wellhead (Allen et al., 2013; Johnson et al., 2022). These studies reported that more efficient emission‐control equipment, and elimination of previously uncontrolled releases from individual wells, helped to reduce produced water‐related air emissions.

***Leaks or Spills into Groundwater***. In many studies, investigators emphasized the importance of comprehensive and consistent monitoring of produced water and its disposal to protect groundwater reservoirs, particularly reservoirs that overlay oil and gas production areas, have natural hydraulic connections to formation water, or can be contaminated by produced‐water leaks from impoundments (Abraham et al., 2023; Drollette et al., 2015; Llewellyn et al., 2015; McMahon et al., 2017; Reilly et al., 2015; Rish & Pfau, 2018; Wang, 2021; Warner, Kresse, et al., 2013; Wright et al., 2019).

In the case of potable drinking water wells, several studies conducted in the Eagle Ford, Fayetteville, Haynesville, and Marcellus shale regions, as well as in California, did not find evidence of produced‐water contamination. Instead, those studies reported contamination from other sources such as animal waste, septic effluent, and road salt, or attributed rapid rates of flushing in the aquifer system as to why contamination may have been minimal (McMahon et al., 2017; Reilly et al., 2015; Warner, Kresse, et al., 2013; Wright et al., 2019). One study analyzed samples from several domestic groundwater wells in Pennsylvania due to reported contamination following the development of five UOGD wells 1–2 km away (Llewellyn et al., 2015). The investigators hypothesized that a leak reported from a produced‐water pit at the nearest UOGD well could have been the source as measurements were consistent with UOGD produced water in the Marcellus region. However, they were unable to fingerprint the contaminant source due to the lack of drilling, pit, or hydraulic fracturing fluid samples from nearby development. Additional data gaps around such investigations include a lack of an up‐to‐date publicly available database of polymer‐lined containment pits for produced water, and limited volume and composition data in the Pennsylvania Department of Environmental Protection's (PADEP) disclosed violation reports for spills (Drollette et al., 2015; Rish & Pfau, 2018).

An additional concern for potential groundwater contamination is produced‐water leakage from abandoned oil wells. Scanlon et al. highlighted several data gaps around confirming evidence for this exposure pathway, including a lack of produced‐water composition data and standardized monitoring around known locations of abandoned wells to assess potential leakages (Scanlon et al., 2021).

Unlined produced‐water disposal ponds designed to passively treat produced water can pose exposure risks by leaking into surrounding or underlying groundwater reservoirs (DiGiulio et al., 2021; DiGiulio & Jackson, 2016; Preston et al., 2019). In the San Joaquin Valley in California, DiGiulio et al. reported elevated levels of TDS and salinity in groundwater around these ponds (DiGiulio et al., 2021). DiGiulio et al. suggested treating produced waters discharged to wastewater disposal ponds to the extent that chemical concentrations are reduced to nonhazardous levels (DiGiulio et al., 2021).

***Leaks or Spills into Surface Water***. Produced‐water leaks and spills into surface water can have long lasting effects on water quality and ecological health both in individual water bodies and at the watershed level (Bonetti et al., 2021; Casey et al., 2022; Cozzarelli et al., 2017, 2021; Lauer et al., 2016; Michaels et al., 2022). Moreover, produced‐water spills contaminating surface water bodies used for drinking water, irrigation water, and recreational activities can potentially pose health risks to nearby communities (Abualfaraj et al., 2018; Torres et al., 2017, 2018).

Produced water from UIC disposal facilities can also leak into surrounding surface water and streambed sediments (Akob et al., 2016; Kassotis et al., 2016). Toxicological analysis at these sites can help ascertain the contamination levels of stream sediment and water, related human exposures, and where in the watershed contaminant levels might be elevated (Orem et al., 2017). Additionally, produced water generated during flowback operations can corrode pipeline infrastructure; these corrosion deposits accumulate in the produced water that is eventually disposed through Class II wells, potentially elevating the toxicity of any accidental environmental releases at UIC facilities (Chilkoor et al., 2018).

Cozzarelli et al. examined a spill event that occurred in January 2015 where 11.4 million liters (or about 3 million gallons) of produced water leaked from a pipeline into Blacktail Creek in the Williston Basin in North Dakota (Cozzarelli et al., 2017, 2021). Cozzarelli et al. reported barium, strontium, and radium activities in downstream sediment 6 months after the spill in concentrations 15 times the upstream concentrations (Cozzarelli et al., 2017). Sediment has the potential to be a long‐term reservoir for constituents even in the surrounding flood plain as the investigators observed persisting barium, strontium, radium, and ammonium levels in sediment 7.2 km downstream from the spill even after 2.5 years. Investigators concluded that the effect of produced‐water constituents on the environment is still poorly understood and highly variable depending on hydrological conditions (Cozzarelli et al., 2021). Biological studies conducted 6‐month to 3 years later revealed continued changes in microbial community structures and ammonium levels attributable to the spill (Farag et al., 2022).

Maloney et al. analyzed spill records from state databases in Colorado, New Mexico, North Dakota, and Pennsylvania; the states chosen for the study were based on data accessibility and the various shale basins that underly the regions (Maloney et al., 2017). From 2005 to 2014, spill rates increased for all states except for Pennsylvania, which began to decrease after an initial increase to plateau in 2009. In Rossi et al., the California Governor's Office of Emergency Services' (CalOES) HazMat database showed that the number of spill incidents and their frequency decreased between 2006 and 2020 and that 16% of the reported incidences of produced‐water spills had documented effects on waterways, with no known impacts to drinking water after CalOES started monitoring drinking water in 2016 (Rossi et al., 2022).

Multiple knowledge gaps affect tracking produced‐water spills, including lack of standardization and transparency in spill reporting and monitoring, exact geographical coordinates of reported spills, information on potential impacts to people and ecosystems, variation in each state's laws and reporting requirements, and unreported spills (Maloney et al., 2017; Rossi et al., 2022).

Attributing changes in surface water quality at the watershed level to environmental releases of produced water is challenging due to the variety of potential pollutant sources, such as natural brine seeps, oil and gas development‐associated wastes besides produced water, coal extraction wastes, mine drainage fluids, agriculture, urban development, road salt, deforestation, and other land use changes (Casey et al., 2022; Johnson et al., 2022; Kassotis et al., 2020; Michaels et al., 2022; Pelak & Sharma, 2014). Some studies in the Marcellus Shale region reported surface water samples with contamination from constituents mimicking produced‐water composition in the region (Harkness et al., 2017; Johnson et al., 2015) or temporal correlations between TDS levels in surface water and drilling phases of oil and gas development in the vicinity (Bonetti et al., 2021). In the same region, Mumford et al. reported that the intensity of oil and gas development in the watershed was independent of changes in stream‐water biology and geochemistry (Mumford et al., 2020).

#### 3.2.2 Authorized Release of Produced Water to the Environment

Methods to treat produced water are often basin‐specific and are primarily aimed at TDS and radium reduction. Treatment of produced water can involve use of a combination of pressure, gravity, and heat to separate the oil, water, and gas phases (Conrad et al., 2020). At the most complex level, treatment of produced water may include advanced treatment methods such as desalination (Conrad et al., 2020). Advanced treatment methods are used to target hazardous compounds to reduce the potential for human exposure; however, treatment byproducts (e.g., radioactive sludge or reject brines from desalination processes) could pose their own threats to human and environmental health.

***Discharges from Wastewater Treatment Facilities***. As UOGD became more prevalent in the United States in the early 2000s, produced water was initially sent to municipal wastewater‐treatment facilities even though these facilities were not designed for this type of wastewater. Investigators reported high concentrations of TDS, salinity, bromide and other ions, which can lead to the formation of disinfection byproducts in the receiving waters for the treatment facility discharges (Burgos et al., 2017; Ferrar et al., 2013; Lauer et al., 2018; Skalak et al., 2013; Van Sice et al., 2018; Warner, Christie, et al., 2013). DBPs are relevant to human and ecological health due to their carcinogenicity and toxicity (Hladik et al., 2014; Huang et al., 2018; Landis et al., 2016; Parker et al., 2014; States et al., 2013; Weaver et al., 2015; Wilson & VanBriesen, 2012). In 2011, the PADEP issued a voluntary request to drilling companies to recycle produced water from UOGD instead of disposing it through municipal wastewater treatment plants. In 2016, EPA issued a mandatory ruling that made the voluntary request law (U.S. Environmental Protection Agency, 2020). While produced water from COGD contain many constituents of concern similar to UOGD (Tasker et al., 2020), it can still be treated by municipal wastewater treatment plants (U.S. Environmental Protection Agency, 2020).

***Discharges to Surface Water and Applications to Land***. Water‐stressed regions in the western United States are utilizing, and considering expansion of, produced water for agriculture and other uses to conserve potable water (Texas Produced Water Consortium, 2022). Furthermore, under the Clean Water Act, minimally treated produced water can be discharged to surface water west of the 98th meridian if it is deemed of “good enough quality” for beneficial use (e.g., crop irrigation, livestock watering, and wildlife propagation). “Good enough quality” can vary greatly by state with a NPDES specific conductance effluent limitation daily maximum of 7,500 µS/cm for produced water discharged to surface water in Wyoming versus 1,000 µS/cm in California for produced water reclaimed for irrigation water (Kondash et al., 2020; McLaughlin, Borch, et al., 2020).

Several investigators sampled and analyzed treated produced water and surface water at the point of discharge in Wyoming, upstream of a drinking water reservoir (McDevitt, McLaughlin, et al., 2020, McDevitt et al., 2024; McLaughlin, Borch, et al., 2020, 2021). Using a gas chromatography‐based nontargeted analysis, McLaughlin et al. detected more than 50 organic chemicals in surface water at the point of discharge (McLaughlin, Blotevogel, et al., 2020). They reported that the chemicals were not specified in effluent limit guidelines of the specific NPDES permit for this discharge, and that few had health thresholds for humans, livestock, and aquatic species. While the regulated concentrations mostly met NPDES limits at the point of discharge, inorganic chemicals exceeded limits downstream due to evaporation, suggesting the need for regulations in arid and semi‐arid regions to account for the effect of climate on the composition of the discharge (McDevitt, McLaughlin, et al., 2020). In a companion study that measured chronic toxicity of this produced‐water release (McLaughlin, Borch, et al., 2020), the research team reported higher mutation rates in yeast cells in the water, which correlated with the concentrations of benzene and radium (known carcinogens) in the discharge stream reported on previously (McLaughlin, Blotevogel, et al., 2020). Minimal acute toxicity and no developmental toxicity were observed. The investigators suggested that such toxicological investigations are necessary to determine potential health impacts to downstream users, especially since reporting requirements for NPDES permits apply to the discharge site only rather than downstream changes in concentration, which are more relevant to adequately protect human health.

McDevitt, McLaughlin, et al. (2021) and McLaughlin et al. (2021) evaluated the potential for wetlands to attenuate potentially harmful constituents in produced water discharged for beneficial use. Wetland systems, both natural and constructed, employ vegetation and soil to naturally treat water through processes like sorption and biodegradation. After analyzing surface water and sediment samples, they reported that, under oxygenated conditions, such systems may reduce dissolved radionuclide and organic chemical additive loads from produced water (McDevitt, McLaughlin, et al., 2021; McLaughlin et al., 2021).

***Irrigation***. Studies examined the effect of produced water treated to various extents used to irrigate crops (Miller et al., 2020; Redmon et al., 2021; Sappington & Rifai, 2018; Sedlacko et al., 2022). The high salinity typically associated with many produced waters can decrease the water infiltration rate of water into soil and impact crop production (Oetjen et al., 2018; U.S. Environmental Protection Agency, 2020). Miller et al. reported reduced soil health and significant changes in microbial community diversity and structure that may shape soil biochemical cycling and yield (Miller et al., 2020). Sedlacko et al. observed metabolite changes in produced water irrigated wheat that indicated plant stress independent of salinity (Sedlacko et al., 2022). Sappington and Rifai observed symptoms of salt stress but saw positive effects on crop germination in cowpeas when irrigated with produced water treated to lower TDS levels (Sappington & Rifai, 2018). Combining field monitoring data of irrigation water, soil, and edible crops with information from state water and soil survey databases, Redmon et al. quantified minimal health risk from the consumption of trace metals in crops irrigated with oil field produced water that is pretreated to reduce salinity (Redmon et al., 2021). Generally, investigators suggested further, large‐scale study to determine long‐term effects on soil, plants, and human health.

***Treatment of Roads***. Farnan et al. analyzed multiple types of road applicants for efficacy as dust suppressants, including ten produced‐water samples from Pennsylvania, as produced water has been used for road treatment in a number of states for the past 70 years (Farnan et al., 2023). They reported road runoff during a rain event as a potential source of radium entering the environment. Exposure to constituents of produced water used to de‐ice and suppress dust on roadways could also occur through deposition of alkaline earth metals such as radium and barium in soil and sediment along roads, and via evaporation in residential areas where people may spend more time on sidewalks and driveways (Bain et al., 2021; Skalak et al., 2013). Additionally, Stallworth et al. reported that produced water used as a dust suppressant was less effective than commercial road maintenance products due to the destabilization of road aggregate with high sodium brines, potentially exposing humans to increased inhalation risks (Stallworth et al., 2021). Researchers suggested further investigation to clarify potential exposures from road treatment, particularly to better understand the actual exposure times for people occupying spaces in proximity to roadways and to consider the composition of produced water spread on roads.

#### 3.2.3 Potentially Exposed Human Populations

Human exposure to produced water can vary depending on the variability of produced‐water composition (Figure 4) and management practices (Figure 1). Populations can be exposed to constituents in produced water by inhaling compounds that evaporate from storage units like impoundments or that are applied as road treatment for dust suppression or de‐icing. Accidental leaks and intentional releases of produced water from wells, storage units, and disposal facilities can contaminate surface water and groundwater, and the construction and maintenance deficiencies such as corroded pipeline infrastructure or improperly sealed well casing can result in spills or leaks from well pad equipment or pipelines (Chilkoor et al., 2018; Li & Carlson, 2014; Scanlon et al., 2021; Ziemkiewicz et al., 2014). Discharges of produced water from treatment facilities to surface water can represent another potential pathway of exposure, especially if the receiving water body is used for recreation or as a source of drinking water or irrigation water. The type and magnitude of exposure would depend on the treatment methodology and efficiency.

Many studies summarized in this review reported evidence of contamination from produced water in various environmental media or the potential for human exposure. However, whether the exposures represent an important health risk is difficult to assess given the unknowns about produced‐water composition and how its constituents migrate, transform, and degrade following release to environments with variable meteorological and hydrological conditions. Of the 236 studies (Figure 2), only seven conducted assessments of human exposure to produced water (Bain et al., 2021; Ma et al., 2019; McLaughlin, Borch, et al., 2020; Soriano et al., 2022; Torres et al., 2017, 2018; Zhang et al., 2015), and five included information characterizing the communities who may be exposed to produced water (Bain et al., 2021; Johnston et al., 2016; Reilly et al., 2015; Silva et al., 2018; Soriano et al., 2022).

Of the studies that examined and distinguished among specific exposed subpopulations, investigators generally reported that populations living near infrastructure such as disposal wells, commuter routes using produced water for deicing, and private residential groundwater wells hypothesized to be contaminated by flowback, tended to have attributes including high poverty rates, low median household income, high proportions of people of color, or be rural (Bain et al., 2021; Johnston et al., 2016; Reilly et al., 2015; Silva et al., 2018).

### 3.3 Addressing Gaps in Understanding About Human Exposure to Produced Water

Oil and gas development will continue for some time (IEA, 2023), so it is important to understand how communities may potentially be exposed to produced water, especially given the growing interest in using produced water outside of the oil field. The 236 publications reviewed here contribute to this understanding, but knowledge gaps remain in how people might be exposed to produced water, for example, if it will be used to supplement freshwater at commercially viable scales or as a source of lithium and other commodities (Kumar et al., 2019; Liu et al., 2023).

Specific knowledge gaps pertain to the analytical methods to detect and measure chemicals in produced water (its characterization made more challenging by its high‐salinity and variable composition), the documentation of environmental releases, the performance of treatment methods required for various end uses, the migration pathways through the environment that can result in human exposure, characterization of exposed populations, and the significance of exposures for health.

The toxicology studies highlighted here exposed animal or cultured cells to a subset of chemicals found in produced water and found a variety of adverse health effects; these results indicate potential health risks in humans. Some investigators noted a particular need to investigate the toxicological effects of chronic and acute exposures to chemical mixtures resulting from produced‐water releases to the environment (Kassotis et al., 2015; Rich & Crosby, 2013; Smalling et al., 2019). An increased understanding of the chemicals and their toxicity in produced water could allow for a more comprehensive exposure pathway analysis to assess health risks associated with exposure to produced water. Epidemiology studies are used to understand how exposure to chemical mixtures such as produced water can affect human health. To date, epidemiology studies examining associations between onshore oil and gas and health outcomes have used exposure metrics incorporating information about distance from or density of wells or estimated exposure to releases via the air pathway. To our knowledge, only one epidemiology study attempted to quantify associations between produced water exposure and health by using “average monthly produced water attributed to a given drilling site over its lifetime” as the exposure metric (Willis et al., 2022). The investigators reported increased odds of congenital anomalies compared to the reference group. An improved understanding of exposure pathways would be useful for designing epidemiology studies to assess potential links between produced water and human health.

The Groundwater Protection Council summarizes information about produced‐water volumes and management that is collected by state and federal agencies, and describes systemic limitations in this documentation, including data availability, completeness, accuracy, quality, and differences in reporting, collection, and categorization (i.e., basic definitions) of handling and disposition (ALL Consulting, 2022; Clark & Veil, 2009; Veil, 2015, 2020). Other investigators identified several gaps in reporting about the composition and release of produced water to the environment, which can limit the ability to identify exposure pathways, and suggested comprehensive, standardized, and accessible reporting (Danforth et al., 2019; Drollette et al., 2015; Ferrar et al., 2013; Li et al., 2016; Llewellyn et al., 2015; Wilson & VanBriesen, 2012).

The curation of a standardized database that tracks and accounts for produced‐water volumes, disposition, spills, and use at the state or even national level could address several limitations towards understanding potential human exposure. Standardized data collection and curation could help to mitigate variations in each state's reporting for produced water and promote transparency around how waste is regulated (Malone et al., 2015). Where data are available, meta‐analyses of events or parameters that lead to spills can inform risk assessment and guide regulations to reduce unintended releases to the environment (Maloney et al., 2017).

In several studies, investigators observed various construction and maintenance deficiencies related to pipelines and storage systems that may lead to environmental releases and potential exposures (DiGiulio & Jackson, 2016; Li et al., 2016; Ziemkiewicz et al., 2014). Patterns related to other practices that may lead to environmental releases could be identified and addressed if more details were included in state and federal records about produced‐water leaks or spills. This detail could include information leading to the incident, exact geographic coordinates of the spill, released volumes, and related monitoring over time to inform source appropriation and assessments of exposure and health risk (Gross et al., 2013; Maloney et al., 2017; Rossi et al., 2022; Torres et al., 2017). Furthermore, such documentation could address the challenge of distinguishing between exposures to produced water and exposures to other environmental releases in locations with a long history of energy development (Rich & Crosby, 2013). Finally, as NPDES‐permitted discharges and land applications of produced water continue or expand, extended monitoring and reporting of composition and volume throughout production, discharge, and use would be helpful for tracking potential exposures at each stage of management (Bain et al., 2021; McLaughlin, Blotevogel, et al., 2020; Redmon et al., 2021; Scanlon et al., 2021).

Addressing these knowledge gaps could allow for the benefits of produced water use to be better realized while simultaneously protecting human health. Additionally, characterizing populations potentially exposed to produced water would have the added benefit of prioritizing the allocation of resources to protect the most vulnerable members of exposed populations.

## Conflict of Interest

The U.S. Geological Survey authors declare they have no actual or potential competing financial interests. The Health Effects Institute authors receive joint funding from the U.S. Environmental Protection Agency and several oil and gas companies, but these sponsors played no role in the development or review of this manuscript.

## Supporting information

Supporting Information S1

## Data Availability

No new data were created or analyzed during this study. Data sharing is not applicable to this article.

## References

[gh270015-bib-0001] Abraham, D. , Liberatore, H. , Aziz, M. T. , Burnett, D. , Cizmas, L. , & Richardson, S. (2023). Impacts of hydraulic fracturing wastewater from oil and gas industries on drinking water: Quantification of 69 disinfection by‐products and calculated toxicity. Science of the Total Environment, 882, 163344. 10.1016/j.scitotenv.2023.163344 37030373

[gh270015-bib-0002] Abualfaraj, N. , Gurian, P. , & Olson, M. (2018). Assessing residential exposure risk from spills of flowback water from Marcellus shale hydraulic fracturing activity. International Journal of Environmental Research and Public Health, 15(4), 727. 10.3390/ijerph15040727 29641504 PMC5923769

[gh270015-bib-0003] Abualfaraj, N. , Gurian, P. L. , & Olson, M. S. (2014). Characterization of Marcellus shale flowback water. Environmental Engineering Science, 31(9), 514–524. 10.1089/ees.2014.0001

[gh270015-bib-0004] Acharya, S. M. , Chakraborty, R. , & Tringe, S. G. (2020). Emerging trends in biological treatment of wastewater from unconventional oil and gas extraction. Frontiers in Microbiology, 11. 10.3389/fmicb.2020.569019 PMC7509137 33013800

[gh270015-bib-0005] Aghababaei, M. , Luek, J. L. , Ziemkiewicz, P. F. , & Mouser, P. J. (2021). Toxicity of hydraulic fracturing wastewater from black shale natural‐gas wells influenced by well maturity and chemical additives. Environmental Sciences: Processes & Impacts, 23(4), 621–632. 10.1039/D1EM00023C 33908986

[gh270015-bib-0006] Akob, D. M. , Cozzarelli, I. M. , Dunlap, D. S. , Rowan, E. L. , & Lorah, M. M. (2015). Organic and inorganic composition and microbiology of produced waters from Pennsylvania shale gas wells. Applied Geochemistry, 60, 116–125. 10.1016/j.apgeochem.2015.04.011

[gh270015-bib-0007] Akob, D. M. , Mumford, A. C. , Orem, W. H. , Engle, M. A. , Klinges, J. G. , Kent, D. B. , & Cozzarelli, I. M. (2016). Wastewater disposal from unconventional oil and gas development degrades stream quality at a West Virginia injection facility. Environmental Science & Technology, 50(11), 5517–5525. 10.1021/Acs.Est.6b00428 27158829

[gh270015-bib-0008] Akyon, B. , McLaughlin, M. , Hernández, F. , Blotevogel, J. , & Bibby, K. (2018). Characterization and biological removal of organic compounds from hydraulic fracturing produced water. Environmental Sciences: Processes & Impacts. 10.1039/C8EM00354H 30451271

[gh270015-bib-0009] Al‐Ghouti, M. A. , Al‐Kaabi, M. A. , Ashfaq, M. Y. , & Da’na, D. A. (2019). Produced water characteristics, treatment and reuse: A review. Journal of Water Process Engineering, 28, 222–239. 10.1016/j.jwpe.2019.02.001

[gh270015-bib-0010] ALL Consulting . (2022). US produced water volumes & management practices in 2021 | Groundwater Protection Council. Retrieved from https://www.gwpc.org/wp‐content/uploads/2021/09/2021_Produced_Water_Volumes.pdf

[gh270015-bib-0011] Allen, D. T. , Torres, V. M. , Thomas, J. , Sullivan, D. W. , Harrison, M. , Hendler, A. , et al. (2013). Measurements of methane emissions at natural gas production sites in the United States. Proceedings of the National Academy of Sciences of the United States of America, 110(44), 17768–17773. 10.1073/pnas.1304880110 24043804 PMC3816463

[gh270015-bib-0012] American Geosciences Institute . (2016). Retrieved from https://www.americangeosciences.org/critical‐issues/faq/what‐produced‐water

[gh270015-bib-0013] Ariana, A. , Rosofsky, A. , Danforth, C. , & Vorhees, D. (2023). Potential human exposures to produced water from onshore oil and gas production (Research Brief No. 5) (p. 51). Health Effects Institute Energy. Retrieved from https://www.heienergy.org/publication/potential‐human‐exposures‐produced‐water‐onshore‐oil‐and‐gas‐production

[gh270015-bib-0014] Bain, D. J. , Cantlay, T. , Garman, B. , & Stolz, J. F. (2021). Oil and gas wastewater as road treatment: Radioactive material exposure implications at the residential lot and block scale. Environmental Research Communications, 3(11), 115008. 10.1088/2515-7620/ac35be

[gh270015-bib-0015] Balise, V. D. , Cornelius‐Green, J. N. , Kassotis, C. D. , Rector, R. S. , Thyfault, J. P. , & Nagel, S. C. (2019). Preconceptional, gestational, and lactational exposure to an unconventional oil and gas chemical mixture alters energy expenditure in adult female mice. Frontiers in Endocrinology, 10. 10.3389/fendo.2019.00323 PMC6540741 31191452

[gh270015-bib-0016] Balise, V. D. , Cornelius‐Green, J. N. , Parmenter, B. , Baxter, S. , Kassotis, C. D. , Rector, R. S. , et al. (2019). Developmental exposure to a mixture of unconventional oil and gas chemicals increased risk‐taking behavior, activity and energy expenditure in aged female mice after a metabolic challenge. Frontiers in Endocrinology, 10, 460. 10.3389/fendo.2019.00460 31402896 PMC6669236

[gh270015-bib-0017] Barnaby, R. J. , Oetting, G. C. , & Gao, G. (2004). Strontium isotopic signatures of oil‐field waters: Applications for reservoir characterization. AAPG Bulletin, 88(12), 1677–1704. 10.1306/07130404002

[gh270015-bib-0018] Bean, J. K. , Bhandari, S. , Bilotto, A. , & Hildebrandt Ruiz, L. (2018). Formation of particulate matter from the oxidation of evaporated hydraulic fracturing wastewater. Environmental Science & Technology, 52(8), 4960–4968. 10.1021/acs.est.7b06009 29596740

[gh270015-bib-0019] Blauch, M. E. , Myers, R. R. , Moore, T. R. , Lipinski, B. A. , & Houston, N. A. (2009). Marcellus shale post‐frac flowback waters – Where is all the salt coming from and what are the implications? Presented at the SPE Eastern Regional Meeting, OnePetro. SPE Eastern Regional Meeting. 10.2118/125740-MS

[gh270015-bib-0020] Blondes, M. S. , Gans, K. D. , Engle, M. A. , Kharaka, Y. K. , Reidy, M. E. , Saraswathula, V. , et al. (2019). U.S. Geological survey national produced waters geochemical database ver. 2.3 [Dataset]. U.S. Geological Survey. 10.5066/F7J964W8

[gh270015-bib-0021] Bonetti, P. , Leuz, C. , & Michelon, G. (2021). Large‐sample evidence on the impact of unconventional oil and gas development on surface waters. Science, 373(6557), 896–902. 10.1126/science.aaz2185 34413233

[gh270015-bib-0022] Booker, A. E. , Borton, M. A. , Daly, R. A. , Welch, S. A. , Nicora, C. D. , Hoyt, D. W. , et al. (2017). Sulfide generation by dominant halanaerobium microorganisms in hydraulically fractured shales. mSphere, 2(4). 10.1128/mspheredirect.00257-17 PMC5497025 28685163

[gh270015-bib-0023] Boulé, L. A. , Chapman, T. J. , Hillman, S. E. , Kassotis, C. D. , O’Dell, C. , Robert, J. , et al. (2018). Developmental exposure to a mixture of 23 chemicals associated with unconventional oil and gas operations alters the immune system of mice. Toxicological Sciences, 163(2), 639–654. 10.1093/toxsci/kfy066 29718478 PMC5974794

[gh270015-bib-0024] Burgos, W. D. , Castillo‐Meza, L. , Tasker, T. L. , Geeza, T. J. , Drohan, P. J. , Liu, X. , et al. (2017). Watershed‐scale impacts from surface water disposal of oil and gas wastewater in western Pennsylvania. Environmental Science & Technology, 51(15), 8851–8860. 10.1021/acs.est.7b01696 28699344

[gh270015-bib-0025] Butkovskyi, A. , Bruning, H. , Kools, S. A. E. , Rijnaarts, H. H. M. , & Van Wezel, A. P. (2017). Organic pollutants in shale gas flowback and produced waters: Identification, potential ecological impact, and implications for treatment strategies. Environmental Science & Technology, 51(9), 4740–4754. 10.1021/acs.est.6b05640 28376616 PMC5415876

[gh270015-bib-0026] Cantlay, T. , Bain, D. J. , Curet, J. , Jack, R. F. , Dickson, B. C. , Basu, P. , & Stolz, J. F. (2020). Determining conventional and unconventional oil and gas well brines in natural sample II: Cation analyses with ICP‐MS and ICP‐OES. Journal of Environmental Science and Health, Part A, 55(1), 11–23. 10.1080/10934529.2019.1666561 31549915

[gh270015-bib-0027] Casey, C. P. , Hartings, M. R. , Knapp, M. A. , Malloy, E. J. , & Knee, K. L. (2022). Characterizing the association between oil and gas development and water quality at a regional scale. Freshwater Science, 41(2), 236–252. 10.1086/719983

[gh270015-bib-0028] Chapman, E. C. , Capo, R. C. , Stewart, B. W. , Kirby, C. S. , Hammack, R. W. , Schroeder, K. T. , & Edenborn, H. M. (2012). Geochemical and strontium isotope characterization of produced waters from Marcellus shale natural gas extraction. Environmental Science & Technology, 46(6), 3545–3553. 10.1021/es204005g 22360406

[gh270015-bib-0029] Chaudhary, B. K. , Sabie, R. , Engle, M. A. , Xu, P. , Willman, S. , & Carroll, K. C. (2019). Spatial variability of produced‐water quality and alternative‐source water analysis applied to the Permian Basin, USA. Hydrogeology Journal, 27(8), 2889–2905. 10.1007/s10040-019-02054-4

[gh270015-bib-0030] Chen, F. , Ma, Z. , Nasrabadi, H. , Chen, B. , Mehana, M. , & Van Wijk, J. (2023). Reuse of produced water from the petroleum industry: Case studies from the intermountain‐west region, USA. Energy & Fuels, 37(5), 3672–3684. 10.1021/acs.energyfuels.2c04000

[gh270015-bib-0031] Chilkoor, G. , Shrestha, N. , Soeder, D. , & Gadhamshetty, V. (2018). Corrosion and environmental impacts during the flowback water disposal associated with the Bakken shale. Corrosion Science, 133, 48–60. 10.1016/j.corsci.2018.01.019

[gh270015-bib-0032] Chittick, E. A. , & Srebotnjak, T. (2017). An analysis of chemicals and other constituents found in produced water from hydraulically fractured wells in California and the challenges for wastewater management. Journal of Environmental Management, 204(Pt 1), 502–509. 10.1016/j.jenvman.2017.09.002 28934673

[gh270015-bib-0033] Clark, C. E. , & Veil, J. A. (2009). Produced water volumes and management practices in the United States. Argonne National Laboratory. Retrieved from https://publications.anl.gov/anlpubs/2009/07/64622.pdf

[gh270015-bib-0034] Cluff, M. A. , Hartsock, A. , MacRae, J. D. , Carter, K. , & Mouser, P. J. (2014). Temporal changes in microbial ecology and geochemistry in produced water from hydraulically fractured Marcellus shale gas wells. Environmental Science & Technology, 48(11), 6508–6517. 10.1021/es501173p 24803059

[gh270015-bib-0035] Conrad, C. L. , Ben Yin, Y. , Hanna, T. , Atkinson, A. J. , Alvarez, P. J. J. , Tekavec, T. N. , et al. (2020). Fit‐for‐purpose treatment goals for produced waters in shale oil and gas fields. Water Research, 173, 115467. 10.1016/j.watres.2020.115467 32006805

[gh270015-bib-0036] Cooper, C. M. , McCall, J. , Stokes, S. C. , McKay, C. , Bentley, M. J. , Rosenblum, J. S. , et al. (2022). Oil and gas produced water reuse: Opportunities, treatment needs, and challenges. ACS ES&T Engineering, 2(3), 347–366. 10.1021/acsestengg.1c00248

[gh270015-bib-0037] Cozzarelli, I. M. , Kent, D. B. , Briggs, M. , Engle, M. A. , Benthem, A. , Skalak, K. J. , et al. (2021). Geochemical and geophysical indicators of oil and gas wastewater can trace potential exposure pathways following releases to surface waters. Science of the Total Environment, 755, 142909. 10.1016/j.scitotenv.2020.142909 33131866

[gh270015-bib-0038] Cozzarelli, I. M. , Kent, D. B. , Croteau, M. N. , Skalak, K. J. , Benthem, A. , Engle, M. A. , et al. (2016). Unconventional oil and gas (UOG) wastewater spills: Persistence of contaminants and environmental health implications. Presented at the GSA Annual Meeting. Geological Society of America Abstracts with Programs, 48, 283704. 10.1130/abs/2016am-283704

[gh270015-bib-0039] Cozzarelli, I. M. , Skalak, K. J. , Kent, D. B. , Engle, M. A. , Benthem, A. , Mumford, A. C. , et al. (2017). Environmental signatures and effects of an oil and gas wastewater spill in the Williston Basin, North Dakota. Science of the Total Environment, 579, 1781–1793. 10.1016/j.scitotenv.2016.11.157 27939081

[gh270015-bib-0040] Crosby, L. M. , Tatu, C. A. , Varonka, M. , Charles, K. M. , & Orem, W. H. (2018). Toxicological and chemical studies of wastewater from hydraulic fracture and conventional shale gas wells. Environmental Toxicology and Chemistry, 37(8), 2098–2111. 10.1002/etc.4146 29630745

[gh270015-bib-0041] Daly, R. A. , Borton, M. A. , Wilkins, M. J. , Hoyt, D. W. , Kountz, D. J. , Wolfe, R. A. , et al. (2016). Microbial metabolisms in a 2.5‐km‐deep ecosystem created by hydraulic fracturing in shales. Nature Microbiology, 1(10), 1–9. 10.1038/nmicrobiol.2016.146 27595198

[gh270015-bib-0042] Danforth, C. , McPartland, J. , Blotevogel, J. , Coleman, N. , Devlin, D. , Olsgard, M. , et al. (2019). Alternative management of oil and gas produced water requires more research on its hazards and risks. Integrated Environmental Assessment and Management, 15(5), 677–682. 10.1002/ieam.4160 30994242

[gh270015-bib-0043] DiGiulio, D. C. , & Jackson, R. B. (2016). Impact to underground sources of drinking water and domestic wells from production well stimulation and completion practices in the Pavillion, Wyoming, field. Environmental Science & Technology, 50(8), 4524–4536. 10.1021/acs.est.5b04970 27022977

[gh270015-bib-0044] DiGiulio, D. C. , Rossi, R. J. , Jaeger, J. M. , Shonkoff, S. B. C. , & Ryan, J. N. (2021). Vulnerability of groundwater resources underlying unlined produced water ponds in the Tulare basin of the San Joaquin Valley, California. Environmental Science & Technology, 55(21), 14782–14794. 10.1021/acs.est.1c02056 34651501

[gh270015-bib-0045] Dresel, P. E. , & Rose, A. W. (2010). Chemistry and origin of oil and gas well brines in western Pennsylvania. Open‐File Report OFOG, 10(01.0).

[gh270015-bib-0046] Drollette, B. D. , Hoelzer, K. , Warner, N. R. , Darrah, T. H. , Karatum, O. , O’Connor, M. P. , et al. (2015). Elevated levels of diesel range organic compounds in groundwater near Marcellus gas operations are derived from surface activities. Proceedings of the National Academy of Sciences of the U S A, 112(43), 13184–13189. 10.1073/Pnas.1511474112 PMC4629325 26460018

[gh270015-bib-0047] Elliott, E. G. , Ettinger, A. S. , Leaderer, B. P. , Bracken, M. B. , & Deziel, N. C. (2016). A systematic evaluation of chemicals in hydraulic‐fracturing fluids and wastewater for reproductive and developmental toxicity. Journal of Exposure Science and Environmental Epidemiology, 27(1), 90–99. 10.1038/jes.2015.81 26732376

[gh270015-bib-0048] Engle, M. A. , Cozzarelli, I. M. , & Smith, B. D. (2014). USGS investigations of water produced during hydrocarbon reservoir development. United States Geological Survey. 10.3133/fs20143104

[gh270015-bib-0049] Farag, A. M. , Harper, D. D. , Cozzarelli, I. M. , Kent, D. B. , Mumford, A. C. , Akob, D. M. , et al. (2022). Using biological responses to monitor freshwater post‐spill conditions over 3 years in Blacktail Creek, north Dakota, USA. Archives of Environmental Contamination and Toxicology, 83, 253–271. 10.1007/s00244-022-00943-6 36129489

[gh270015-bib-0050] Farnan, J. , Vanden Heuvel, J. P. , Dorman, F. L. , Warner, N. R. , & Burgos, W. D. (2023). Toxicity and chemical composition of commercial road palliatives versus oil and gas produced waters. Environmental Pollution, 334, 122184. 10.1016/j.envpol.2023.122184 37453689

[gh270015-bib-0051] Ferrar, K. J. , Michanowicz, D. R. , Christen, C. L. , Mulcahy, N. , Malone, S. L. , & Sharma, R. K. (2013). Assessment of effluent contaminants from three facilities discharging Marcellus shale wastewater to surface waters in Pennsylvania. Environmental Science & Technology, 47(7), 3472–3481. 10.1021/es301411q 23458378

[gh270015-bib-0052] Gallegos, T. J. , Doolan, C. , Caldwell, R. , Engle, M. A. , Varonka, M. , Birdwell, J. , et al. (2021). Insights on geochemical, isotopic, and volumetric compositions of produced water from hydraulically fractured Williston Basin oil wells. Environmental Science & Technology, 55(14), 10025–10034. 10.1021/acs.est.0c06789 34197090

[gh270015-bib-0053] Geeza, T. J. , Gillikin, D. P. , McDevitt, B. , Van Sice, K. , & Warner, N. R. (2018). Accumulation of Marcellus formation oil and gas wastewater metals in freshwater mussel shells. Environmental Science & Technology, 52(18), 10883–10892. 10.1021/acs.est.8b02727 30179464

[gh270015-bib-0054] Gieg, L. M. , Davidova, I. A. , Duncan, K. E. , & Suflita, J. M. (2010). Methanogenesis, sulfate reduction and crude oil biodegradation in hot alaskan oilfields. Environmental Microbiology, 12(11), 3074–3086. 10.1111/j.1462-2920.2010.02282.x 20602630

[gh270015-bib-0055] Goldberg, R. B. , & Griffith, E. M. (2017). Strontium isotopes as a potential fingerprint of total dissolved solids associated with hydraulic‐fracturing activities in the Barnett Shale, Texas. Environmental Geosciences, 24(4), 151–165. 10.1306/eg.06191716501

[gh270015-bib-0056] Gregory, K. B. , Vidic, R. D. , & Dzombak, D. A. (2011). Water management challenges associated with the production of shale gas by hydraulic fracturing. Elements, 7(3), 181–186. 10.2113/gselements.7.3.181

[gh270015-bib-0057] Gross, S. A. , Avens, H. J. , Banducci, A. M. , Sahmel, J. , Panko, J. M. , & Tvermoes, B. E. (2013). Analysis of BTEX groundwater concentrations from surface spills associated with hydraulic fracturing operations. Journal of the Air & Waste Management Association, 63(4), 424–432. 10.1080/10962247.2012.759166 23687727

[gh270015-bib-0058] Groundwater Protection Council . (2023). Produced water report: Regulations & practice updates. Retrieved from https://www.gwpc.org/wp‐content/uploads/2023/06/2023‐Produced‐Water‐Report‐Update‐FINAL‐REPORT.pdf

[gh270015-bib-0059] Harkness, J. S. , Darrah, T. H. , Warner, N. R. , Whyte, C. J. , Moore, M. T. , Millot, R. , et al. (2017). The geochemistry of naturally occurring methane and saline groundwater in an area of unconventional shale gas development. Geochimica et Cosmochimica Acta, 208, 302–334. 10.1016/j.gca.2017.03.039

[gh270015-bib-0060] Hayes, T. (2009). Sampling and analysis of water streams associated with the development of Marcellus shale gas. Des Plains, IL. Retrieved from https://www.water‐research.net/naturalgasPA/pdffiles/MSCommission‐Report.pdf

[gh270015-bib-0061] He, C. , Zhang, T. , & Vidic, R. D. (2016). Co‐treatment of abandoned mine drainage and Marcellus Shale flowback water for use in hydraulic fracturing. Water Research, 104, 425–431. 10.1016/j.watres.2016.08.030 27579871

[gh270015-bib-0062] HEI Energy Research Committee . (2020). Human exposure to unconventional oil and gas development: A literature survey for research planning. Communication 1. Health Effects Institute Energy. Retrieved from https://www.heienergy.org/publication/human‐exposure‐unconventional‐oil‐and‐gas‐development‐literature‐survey‐research

[gh270015-bib-0063] Hildenbrand, Z. L. , Santos, I. C. , Liden, T. , Carlton, D. D. , Varona‐Torres, E. , Martin, M. S. , et al. (2018). Characterizing variable biogeochemical changes during the treatment of produced oilfield waste. Science of the Total Environment, 634, 1519–1529. 10.1016/j.scitotenv.2018.03.388 29710650

[gh270015-bib-0064] Hladik, M. L. , Focazio, M. J. , & Engle, M. (2014). Discharges of produced waters from oil and gas extraction via wastewater treatment plants are sources of disinfection by‐products to receiving streams. Science of the Total Environment, 466, 1085–1093. 10.1016/j.scitotenv.2013.08.008 23994821

[gh270015-bib-0065] Hossack, B. R. , Smalling, K. L. , Anderson, C. W. , Preston, T. M. , Cozzarelli, I. M. , & Ken Honeycutt, R. (2018). Effects of persistent energy‐related brine contamination on Amphibian abundance in national wildlife refuge wetlands. Biological Conservation, 228, 36–43. 10.1016/j.biocon.2018.10.007

[gh270015-bib-0066] Huang, K. Z. , Tang, H. L. , & Xie, Y. F. F. (2018). Impacts of shale gas production wastewater on disinfection byproduct formation: An investigation from a non‐bromide perspective. Water Research, 144, 656–664. 10.1016/j.watres.2018.07.048 30096691

[gh270015-bib-0067] IEA . (2023). World energy Outlook 2023 – Analysis. Retrieved from https://www.iea.org/reports/world‐energy‐outlook‐2023/executive‐summary

[gh270015-bib-0068] Jiang, W. , Pokharel, B. , Lin, L. , Cao, H. , Carroll, K. C. , Zhang, Y. , et al. (2021). Analysis and prediction of produced water quantity and quality in the permian basin using machine learning techniques. Science of the Total Environment, 801, 149693. 10.1016/j.scitotenv.2021.149693 34467907

[gh270015-bib-0069] Jiang, W. , Xu, X. , Hall, R. , Zhang, Y. , Carroll, K. C. , Ramos, F. , et al. (2022). Characterization of produced water and surrounding surface water in the permian basin, the United States. Journal of Hazardous Materials, 430, 128409. 10.1016/j.jhazmat.2022.128409 35149501

[gh270015-bib-0070] Johnson, D. , Clark, N. , Heltzel, R. , Darzi, M. , Footer, T. L. , Herndon, S. , & Thoma, E. D. (2022). Methane emissions from oil and gas production sites and their storage tanks in West Virginia. Atmospheric Environment X, 16, 100193. 10.1016/j.aeaoa.2022.100193 PMC10116818 37091901

[gh270015-bib-0071] Johnson, J. D. , Graney, J. R. , Capo, R. C. , & Stewart, B. W. (2015). Identification and quantification of regional brine and road salt sources in watersheds along the New York/Pennsylvania Border, USA. Applied Geochemistry, 60, 37–50. 10.1016/j.apgeochem.2014.08.002

[gh270015-bib-0072] Johnston, J. E. , Werder, E. , & Sebastian, D. (2016). Wastewater disposal wells, fracking, and environmental injustice in southern Texas. American Journal of Public Health, 106(3), 550–556. 10.2105/AJPH.2015.303000 26794166 PMC4816143

[gh270015-bib-0073] Jubb, A. M. , Engle, M. A. , Chenault, J. M. , Blondes, M. S. , Danforth, C. G. , Doolan, C. , et al. (2020). Direct trace element determination in oil and gas produced waters with inductively coupled plasma‐optical emission spectrometry: Advantages of high‐salinity tolerance. Geostandards and Geoanalytical Research, 44(2), 385–397. 10.1111/ggr.12316

[gh270015-bib-0074] Kashani, M. , Engle, M. A. , Kent, D. B. , Gregston, T. , Cozzarelli, I. M. , Mumford, A. C. , et al. (2024). Illegal dumping of oil and gas wastewater alters arid soil microbial communities. Applied and Environmental Microbiology, 90(2), 014900‐23. 10.1128/aem.01490-23 PMC10880632 38294246

[gh270015-bib-0075] Kassotis, C. D. , Bromfield, J. J. , Klemp, K. C. , Meng, C. X. , Wolfe, A. , Zoeller, R. T. , et al. (2016). Adverse reproductive and developmental health outcomes following prenatal exposure to a hydraulic fracturing chemical mixture in female C57Bl/6 mice. Endocrinology, 157(9), 3469‐81. 10.1210/en.2016-1242 PMC5393361 27560547

[gh270015-bib-0076] Kassotis, C. D. , Harkness, J. S. , Vo, P. H. , Vu, D. C. , Hoffman, K. , Cinnamon, K. M. , et al. (2020). Endocrine disrupting activities and geochemistry of water resources associated with unconventional oil and gas activity. Science of the Total Environment, 748, 142236. 10.1016/j.scitotenv.2020.142236 33039138 PMC7772064

[gh270015-bib-0077] Kassotis, C. D. , Klemp, K. C. , Vu, D. C. , Lin, C.‐H. , Meng, C.‐X. , Besch‐Williford, C. L. , et al. (2015). Endocrine‐Disrupting activity of hydraulic fracturing chemicals and adverse health outcomes after prenatal exposure in male mice. Endocrinology, 156(12), 4458–4473. 10.1210/en.2015-1375 26465197

[gh270015-bib-0078] Kassotis, C. D. , Nagel, S. C. , & Stapleton, H. M. (2018b). Unconventional oil and gas chemicals and wastewater‐impacted water samples promote adipogenesis via PPARγ‐dependent and independent mechanisms in 3T3‐L1 cells. Science of the Total Environment, 640–641, 1601–1610. 10.1016/j.scitotenv.2018.05.030 PMC6197861 29937353

[gh270015-bib-0079] Kassotis, C. D. , Tillitt, D. E. , Davis, J. W. , Hormann, A. M. , & Nagel, S. C. (2014). Estrogen and androgen receptor activities of hydraulic fracturing chemicals and surface and ground water in a drilling‐dense region. Endocrinology, 155(3), 897–907. 10.1210/en.2013-169 24424034

[gh270015-bib-0080] Kassotis, C. D. , Vu, D. C. , Vo, P. H. , Lin, C.‐H. , Cornelius‐Green, J. N. , Patton, S. , & Nagel, S. C. (2018a). Endocrine‐Disrupting activities and organic contaminants associated with oil and gas operations in Wyoming groundwater. Archives of Environmental Contamination and Toxicology, 75(2), 247–258. 10.1007/s00244-018-0521-2 29623359

[gh270015-bib-0081] Kharaka, Y. K. , Thordsen, J. J. , Conaway, C. H. , & Thomas, R. B. (2013). The energy‐water Nexus: Potential groundwater‐quality degradation associated with production of shale gas. Procedia Earth and Planetary Science, 7, 417–422. 10.1016/j.proeps.2013.03.132

[gh270015-bib-0082] Kim, S. , Sick, B. , Omur‐Ozbek, P. , & Carlson, K. H. (2019). Investigating the influence of hydraulic fracturing fluid type and well age on produced water quality: Chemical composition, and treatment and reuse challenges. Desalination and Water Treatment, 146, 39–56. 10.5004/dwt.2019.23665

[gh270015-bib-0083] Kim, S. Y. , Omur‐Ozbek, P. , Dhanasekar, A. , Prior, A. , & Carlson, K. (2016). Temporal analysis of flowback and produced water composition from shale oil and gas operations: Impact of frac fluid characteristics. Journal of Petroleum Science and Engineering, 147, 202–210. 10.1016/j.petrol.2016.06.019

[gh270015-bib-0084] Knierim, K. J. , Blondes, M. S. , Masterson, A. , Freeman, P. , McDevitt, B. , Herzberg, A. , et al. (2024). Evaluation of the lithium resource in the Smackover Formation brines of southern Arkansas using machine learning. Science Advances, 10(39), eadp8149. 10.1126/sciadv.adp8149 39331718 PMC11430454

[gh270015-bib-0085] Kondash, A. J. , Redmon, J. H. , Lambertini, E. , Feinstein, L. , Weinthal, E. , Cabrales, L. , & Vengosh, A. (2020). The impact of using low‐saline oilfield produced water for irrigation on water and soil quality in California. Science of the Total Environment, 733, 139392. 10.1016/j.scitotenv.2020.139392 32446094

[gh270015-bib-0086] Kumar, A. , Fukuda, H. , Hatton, T. A. , & Lienhard, J. H. V. (2019). Lithium recovery from oil and gas produced water: A need for a growing energy industry. ACS Energy Letters, 4(6), 1471–1474. 10.1021/acsenergylett.9b00779

[gh270015-bib-0087] Landis, M. S. , Kamal, A. S. , Kovalcik, K. D. , Croghan, C. , Norris, G. A. , & Bergdale, A. (2016). The impact of commercially treated oil and gas produced water discharges on bromide concentrations and modeled brominated trihalomethane disinfection byproducts at two downstream municipal drinking water plants in the upper allegheny river, Pennsylvania, USA. Science of the Total Environment, 542, 505–520. 10.1016/j.scitotenv.2015.10.074 26520274

[gh270015-bib-0088] Lauer, N. E. , Harkness, J. S. , & Vengosh, A. (2016). Brine spills associated with unconventional oil development in North Dakota. Environmental Science & Technology, 50(10), 5389–5397. 10.1021/acs.est.5b06349 27119384

[gh270015-bib-0089] Lauer, N. E. , Warner, N. R. , & Vengosh, A. (2018). Sources of radium accumulation in stream sediments near disposal sites in Pennsylvania: Implications for disposal of conventional oil and gas wastewater. Environmental Science & Technology, 52(3), 955–962. 10.1021/acs.est.7b04952 29300469

[gh270015-bib-0090] Lester, Y. , Ferrer, I. , Thurman, E. M. , Sitterley, K. A. , Korak, J. A. , Aiken, G. , & Linden, K. G. (2015). Characterization of hydraulic fracturing flowback water in Colorado: Implications for water treatment. Science of the Total Environment, 512–513(0), 637–644. 10.1016/j.scitotenv.2015.01.043 25658325

[gh270015-bib-0091] Li, H. , & Carlson, K. H. (2014). Distribution and origin of groundwater methane in the wattenberg oil and gas field of northern Colorado. Environmental Science & Technology, 48(3), 1484–1491. 10.1021/es404668b 24456231

[gh270015-bib-0092] Li, H. , Son, J.‐H. , & Carlson, K. H. (2016). Concurrence of aqueous and gas phase contamination of groundwater in the wattenberg oil and gas field of northern Colorado. Water Research, 88, 458–466. 10.1016/j.watres.2015.10.031 26519629

[gh270015-bib-0093] Liang, R. , Davidova, I. A. , Marks, C. R. , Stamps, B. W. , Harriman, B. H. , Stevenson, B. S. , et al. (2016). Metabolic capability of a predominant halanaerobium sp. in hydraulically fractured gas wells and its implication in pipeline corrosion. Frontiers in Microbiology, 7. 10.3389/fmicb.2016.00988 PMC4916785 27446028

[gh270015-bib-0094] Liden, T. , Hildenbrand, Z. L. , Sanchez‐Rosario, R. , & Schug, K. A. (2022). Characterizing various produced waters from shale energy extraction within the context of reuse. Energies, 15(13), 4521. 10.3390/en15134521

[gh270015-bib-0095] Lipus, D. , Roy, D. , Khan, E. , Ross, D. , Vikram, A. , Gulliver, D. , et al. (2018). Microbial communities in Bakken region produced water. FEMS Microbiology Letters, 365(12). 10.1093/femsle/fny107 29688457

[gh270015-bib-0096] Lipus, D. , Vikram, A. , Ross, D. , Bain, D. , Gulliver, D. , Hammack, R. , & Bibby, K. (2017). Predominance and metabolic potential of halanaerobium in produced water from hydraulically fractured Marcellus shale wells. Applied and Environmental Microbiology, 83(8). 10.1128/aem.02659-16 PMC5377500 28159795

[gh270015-bib-0097] Liu, Q. , Yang, P. , Tu, W. , Sun, H. , Li, S. , & Zhang, Y. (2023). Lithium recovery from oil and gas produced water: Opportunities, challenges, and future outlook. Journal of Water Process Engineering, 55, 104148. 10.1016/j.jwpe.2023.104148

[gh270015-bib-0098] Llewellyn, G. T. , Dorman, F. , Westland, J. L. , Yoxtheimer, D. , Grieve, P. , Sowers, T. , et al. (2015). Evaluating a groundwater supply contamination incident attributed to Marcellus Shale gas development. Proceedings of the National Academy of Sciences of the U S A, 112(20), 6325–6330. 10.1073/pnas.1420279112 PMC4443362 25941400

[gh270015-bib-0099] Luek, J. L. , & Gonsior, M. (2017). Organic compounds in hydraulic fracturing fluids and wastewaters: A review. Water Research, 123, 536–548. 10.1016/j.watres.2017.07.012 28697484

[gh270015-bib-0100] Ma, L. , Hurtado, A. , Eguilior, S. , & Llamas Borrajo, J. F. (2019). Forecasting concentrations of organic chemicals in the vadose zone caused by spills of hydraulic fracturing wastewater. Science of the Total Environment, 696, 133911. 10.1016/j.scitotenv.2019.133911 31442724

[gh270015-bib-0101] Ma, L. , Hurtado, A. , Eguilior, S. , & Llamas Borrajo, J. F. (2022). Exposure risk assessment to organic compounds based on their concentrations in return water from shale gas developments. Science of the Total Environment, 822, 153586. 10.1016/j.scitotenv.2022.153586 35122853

[gh270015-bib-0102] Mackey, J. , Bain, D. J. , Lackey, G. , Gardiner, J. , Gulliver, D. , & Kutchko, B. (2024). Estimates of lithium mass yields from produced water sourced from the Devonian‐aged Marcellus Shale. Scientific Reports, 14(1), 8813. 10.1038/s41598-024-58887-x 38627528 PMC11021401

[gh270015-bib-0103] Macpherson, G. L. (2015). Lithium in fluids from Paleozoic‐aged reservoirs, appalachian plateau region, USA. Applied Geochemistry, 60, 72–77. 10.1016/j.apgeochem.2015.04.013

[gh270015-bib-0104] Malone, S. , Kelso, M. , Auch, T. , Edelstein, K. , Ferrar, K. , & Jalbert, K. (2015). Data inconsistencies from states with unconventional oil and gas activity. J Environ Sci Health A Tox Hazard Subst Environ Eng, 50(5), 501–510. 10.1080/10934529.2015.992678 25734825

[gh270015-bib-0105] Maloney, K. O. , Baruch‐Mordo, S. , Patterson, L. A. , Nicot, J. P. , Entrekin, S. A. , Fargione, J. E. , et al. (2017). Unconventional oil and gas spills: Materials, volumes, and risks to surface waters in four states of the U.S. The Science of the Total Environment, 581–582, 369–377. 10.1016/j.scitotenv.2016.12.142 28043701

[gh270015-bib-0106] McDevitt, B. , Cavazza, M. , Beam, R. , Cavazza, E. , Burgos, W. D. , Li, L. , & Warner, N. R. (2020). Maximum removal efficiency of barium, strontium, radium, and sulfate with optimum AMD‐Marcellus flowback mixing ratios for beneficial use in the northern Appalachian Basin. Environmental Science & Technology, 54(8), 4829–4839. 10.1021/acs.est.9b07072 32250106

[gh270015-bib-0107] McDevitt, B. , Geeza, T. J. , Gillikin, D. P. , & Warner, N. R. (2021). Freshwater mussel soft tissue incorporates strontium isotopic signatures of oil and gas produced water. ACS ES&T Water, 1(9), 2046–2056. 10.1021/acsestwater.1c00135

[gh270015-bib-0108] McDevitt, B. , Jubb, A. M. , Varonka, M. S. , Blondes, M. S. , Engle, M. A. , Gallegos, T. J. , & Shelton, J. L. (2022). Dissolved organic matter within oil and gas associated wastewaters from U.S. unconventional petroleum plays: Comparisons and consequences for disposal and reuse. Science of the Total Environment, 838, 156331. 10.1016/j.scitotenv.2022.156331 35640759

[gh270015-bib-0109] McDevitt, B. , McLaughlin, M. C. , Blotevogel, J. , Borch, T. , & Warner, N. R. (2021). Oil & Gas produced water retention ponds as potential passive treatment for radium removal and beneficial reuse. Environmental Sciences: Processes & Impacts, 23(3), 501–518. 10.1039/D0EM00413H 33877214

[gh270015-bib-0110] McDevitt, B. , McLaughlin, M. C. , Vinson, D. S. , Geeza, T. J. , Blotevogel, J. , Borch, T. , & Warner, N. R. (2020). Isotopic and element ratios fingerprint salinization impact from beneficial use of oil and gas produced water in the Western U.S. Science of the Total Environment, 716, 137006. 10.1016/j.scitotenv.2020.137006 32069772

[gh270015-bib-0111] McDevitt, B. , Tasker, T. L. , Coyte, R. , Blondes, M. S. , Stewart, B. W. , Capo, R. C. , et al. (2024). Utica/Point Pleasant brine isotopic compositions (δ7Li, δ11B, δ138Ba) elucidate mechanisms of lithium enrichment in the Appalachian Basin. Science of the Total Environment, 947, 174588. 10.1016/j.scitotenv.2024.174588 38981550

[gh270015-bib-0112] McLaughlin, M. C. , Blotevogel, J. , Watson, R. A. , Schell, B. , Blewett, T. A. , Folkerts, E. J. , et al. (2020). Mutagenicity assessment downstream of oil and gas produced water discharges intended for agricultural beneficial reuse. Science of the Total Environment, 715, 136944. 10.1016/j.scitotenv.2020.136944 32014773 PMC7243347

[gh270015-bib-0113] McLaughlin, M. C. , Borch, T. , McDevitt, B. , Warner, N. R. , & Blotevogel, J. (2020). Water quality assessment downstream of oil and gas produced water discharges intended for beneficial reuse in arid regions. The Science of the Total Environment, 713, 136607. 10.1016/j.scitotenv.2020.136607 31955100

[gh270015-bib-0114] McLaughlin, M. C. , McDevitt, B. , Miller, H. , Amundson, K. K. , Wilkins, M. J. , Warner, N. R. , et al. (2021). Constructed wetlands for polishing oil and gas produced water releases. Environmental Sciences: Processes & Impacts, 23(12), 1961–1976. 10.1039/D1EM00311A 34723304

[gh270015-bib-0115] McLimans, C. J. , Shelledy, K. , Conrad, W. , Prendergast, K. , Le, A. N. , Grant, C. J. , & Buonaccorsi, V. P. (2022). Potential biomarkers of endocrine and habitat disruption identified via RNA‐Seq in Salvelinus fontinalis with proximity to fracking operations in Pennsylvania headwater stream ecosystems. Ecotoxicology, 31(6), 1044–1055. 10.1007/s10646-022-02564-0 35834075

[gh270015-bib-0116] McMahon, P. B. , Barlow, J. R. B. , Engle, M. A. , Belitz, K. , Ging, P. B. , Hunt, A. G. , et al. (2017). Methane and benzene in drinking‐water wells overlying the Eagle Ford, Fayetteville, and Haynesville shale hydrocarbon production areas. Environmental Science & Technology, 51(12), 6727–6734. 10.1021/acs.est.7b00746 28562061

[gh270015-bib-0117] McMahon, P. B. , Kulongoski, J. T. , Vengosh, A. , Cozzarelli, I. M. , Landon, M. K. , Kharaka, Y. K. , et al. (2018). Regional patterns in the geochemistry of oil‐field water, southern San Joaquin Valley, California, USA. Applied Geochemistry, 98, 127–140. 10.1016/j.apgeochem.2018.09.015

[gh270015-bib-0118] Michaels, R. , Eliason, K. , Kuzniar, T. , Petty, J. T. , Strager, M. P. , Ziemkiewicz, P. F. , & Morrissey, E. (2022). Microbial communities reveal impacts of unconventional oil and gas development on headwater streams. Water Research, 212, 118073. 10.1016/j.watres.2022.118073 35091219

[gh270015-bib-0119] Miller, H. , Dias, K. , Hare, H. , Borton, M. A. , Blotevogel, J. , Danforth, C. , et al. (2020). Reusing oil and gas produced water for agricultural irrigation: Effects on soil health and the soil microbiome. Science of the Total Environment, 722, 137888. 10.1016/j.scitotenv.2020.137888 32208259

[gh270015-bib-0120] Mumford, A. C. , Maloney, K. O. , Akob, D. M. , Nettemann, S. , Proctor, A. , Ditty, J. , et al. (2020). Shale gas development has limited effects on stream biology and geochemistry in a gradient‐based, multiparameter study in Pennsylvania. Proceedings of the National Academy of Sciences, 117(7), 201911458. 10.1073/pnas.1911458117 PMC7035526 32015108

[gh270015-bib-0121] Murali Mohan, A. , Hartsock, A. , Bibby, K. J. , Hammack, R. W. , Vidic, R. D. , & Gregory, K. B. (2013). Microbial community changes in hydraulic fracturing fluids and produced water from shale gas extraction. Environmental Science & Technology, 47(22), 13141–13150. 10.1021/es402928b 24088205

[gh270015-bib-0122] Murali Mohan, A. , Hartsock, A. , Hammack, R. W. , Vidic, R. D. , & Gregory, K. B. (2013). Microbial communities in flowback water impoundments from hydraulic fracturing for recovery of shale gas. FEMS Microbiology Ecology, 86(3), 567–580. 10.1111/1574-6941.12183 23875618

[gh270015-bib-0123] Nagel, S. C. , Kassotis, C. D. , Vandenberg, L. N. , Lawrence, B. P. , Robert, J. , & Balise, V. D. (2020). Developmental exposure to a mixture of unconventional oil and gas chemicals: A review of effects on adult health, behavior, and disease. Molecular and Cellular Endocrinology, 513, 110722. 10.1016/j.mce.2020.110722 32147523 PMC7539678

[gh270015-bib-0124] Nell, M. , & Helbling, D. E. (2019). Exploring matrix effects and quantifying organic additives in hydraulic fracturing associated fluids using liquid chromatography electrospray ionization mass spectrometry. Environmental Sciences: Processes & Impacts, 21(2), 195–205. 10.1039/C8EM00135A 29790879

[gh270015-bib-0125] New Mexico Environment Department . (2024). New Mexico produced water. Retrieved from https://www.env.nm.gov/new‐mexico‐produced‐water/

[gh270015-bib-0126] Nicot, J.‐P. , Gherabati, A. , Darvari, R. , & Mickler, P. (2018). Salinity reversal and water freshening in the Eagle Ford shale, Texas, USA. ACS Earth and Space Chemistry, 2(11), 1087–1094. 10.1021/acsearthspacechem.8b00095

[gh270015-bib-0127] Oetjen, K. , Blotevogel, J. , Borch, T. , Ranville, J. F. , & Higgins, C. P. (2018). Simulation of a hydraulic fracturing wastewater surface spill on agricultural soil. The Science of the Total Environment, 645, 229–234. 10.1016/j.scitotenv.2018.07.043 30029106

[gh270015-bib-0128] Oetjen, K. , Giddings, C. G. S. , McLaughlin, M. , Nell, M. , Blotevogel, J. , Helbling, D. E. , et al. (2017). Emerging analytical methods for the characterization and quantification of organic contaminants in flowback and produced water. Trends in Environmental Analytical Chemistry, 15, 12–23. 10.1016/j.teac.2017.07.002

[gh270015-bib-0129] Oetjen, K. , & Thomas, L. (2016). Volatile and semi‐volatile organic compound patterns in flowback waters from fracturing sites within the Marcellus Shale. Environmental Earth Sciences, 75(12), 1–10. 10.1007/s12665-016-5847-3

[gh270015-bib-0130] Ogbuji, B. , Nnanna, A. G. A. , Engle, M. , & Amesquita, R. (2022). Compositional analysis of conventional and unconventional permian basin‐produced waters: A simple tool for predicting major ion composition. SPE Production & Operations, 37(03), 383–396. 10.2118/209599-PA

[gh270015-bib-0131] Orem, W. , Tatu, C. , Varonka, M. , Lerch, H. , Bates, A. , Engle, M. , et al. (2014). Organic substances in produced and formation water from unconventional natural gas extraction in coal and shale. International Journal of Coal Geology, 126, 20–31. 10.1016/j.coal.2014.01.003

[gh270015-bib-0132] Orem, W. , Varonka, M. , Crosby, L. , Haase, K. , Loftin, K. , Hladik, M. , et al. (2017). Organic geochemistry and toxicology of a stream impacted by unconventional oil and gas wastewater disposal operations. Applied Geochemistry, 80, 155–167. 10.1016/j.apgeochem.2017.02.016

[gh270015-bib-0133] Osborn, S. G. , & McIntosh, J. C. (2010). Chemical and isotopic tracers of the contribution of microbial gas in Devonian organic‐rich shales and reservoir sandstones, northern Appalachian Basin. Applied Geochemistry, 25(3), 456–471. 10.1016/j.apgeochem.2010.01.001

[gh270015-bib-0134] Ouyang, B. , Renock, D. J. , Ajemigbitse, M. A. , Van Sice, K. , Warner, N. R. , Landis, J. D. , & Feng, X. (2019). Radium in hydraulic fracturing wastewater: Distribution in suspended solids and implications to its treatment by sulfate co‐precipitation. Environmental Sciences: Processes & Impacts, 21(2), 339–351. 10.1039/C8EM00311D 30516236

[gh270015-bib-0135] Parker, K. M. , Zeng, T. , Harkness, J. , Vengosh, A. , & Mitch, W. A. (2014). Enhanced formation of disinfection byproducts in shale gas wastewater‐impacted drinking water supplies. Environmental Science & Technology, 48(19), 11161–11169. 10.1021/es5028184 25203743

[gh270015-bib-0136] Patnode, K. A. , Hittle, E. , Anderson, R. , Zimmerman, L. , & Fulton, J. (2015). Effects of high salinity wastewater discharges on unionid mussels in the Allegheny river, Pennsylvania. Journal of Fish and Wildlife Management, 6(1), 55–70. 10.3996/052013-jfwm-033

[gh270015-bib-0137] Pelak, A. J. , & Sharma, S. (2014). Surface water geochemical and isotopic variations in an area of accelerating Marcellus Shale gas development. Environmental Pollution, 195, 91–100. 10.1016/j.envpol.2014.08.016 25201226

[gh270015-bib-0138] Peterman, Z. E. , Thamke, J. , Futa, K. , & Preston, T. (2012). Strontium isotope systematics of mixing groundwater and oil‐field brine at Goose Lake in northeastern Montana, USA. Applied Geochemistry, 27(12), 2403–2408. 10.1016/j.apgeochem.2012.08.004

[gh270015-bib-0139] Phan, T. T. , Hakala, J. A. , & Bain, D. J. (2018). Influence of colloids on metal concentrations and radiogenic strontium isotopes in groundwater and oil and gas‐produced waters. Applied Geochemistry, 95, 85–96. 10.1016/j.apgeochem.2018.05.018

[gh270015-bib-0140] Piotrowski, P. K. , Tasker, T. L. , Geeza, T. J. , McDevitt, B. , Gillikin, D. P. , Warner, N. R. , & Dorman, F. L. (2020). Forensic tracers of exposure to produced water in freshwater mussels: A preliminary assessment of Ba, Sr, and cyclic hydrocarbons. Scientific Reports, 10(1), 15416. 10.1038/s41598-020-72014-6 32963276 PMC7508860

[gh270015-bib-0141] Preston, T. M. , Anderson, C. W. , Thamke, J. N. , Hossack, B. R. , Skalak, K. J. , & Cozzarelli, I. M. (2019). Predicting attenuation of salinized surface‐ and groundwater‐resources from legacy energy development in the Prairie Pothole Region. Science of the Total Environment, 690, 522–533. 10.1016/j.scitotenv.2019.06.428 31301493

[gh270015-bib-0142] Railroad Commission of Texas . (2024). Produced water beneficial reuse: Framework for pilot study authorization. Retrieved from https://www.rrc.texas.gov/media/nznn2wsj/240108‐produced‐water‐framework‐final.pdf

[gh270015-bib-0143] Redmon, J. H. , Kondash, A. J. , Womack, D. , Lillys, T. , Feinstein, L. , Cabrales, L. , et al. (2021). Is food irrigated with oilfield‐produced water in the California central valley Safe to eat? A probabilistic human health risk assessment evaluating trace metals exposure. Risk Analysis, 41(8), 1463–1477. 10.1111/risa.13641 33336407 PMC8519025

[gh270015-bib-0144] Reilly, D. , Singer, D. , Jefferson, A. , & Eckstein, Y. (2015). Identification of local groundwater pollution in northeastern Pennsylvania: Marcellus flowback or not? Environmental Earth Sciences, 73(12), 8097–8109. 10.1007/s12665-014-3968-0

[gh270015-bib-0145] Rich, A. L. , & Crosby, E. C. (2013). Analysis of reserve pit sludge from unconventional natural gas hydraulic fracturing and drilling operations for the presence of technologically enhanced naturally occurring radioactive material (TENORM). New Solutions, 23(1), 117–135. 10.2190/NS.23.1.h 23552651

[gh270015-bib-0146] Rish, W. R. , & Pfau, E. J. (2018). Bounding analysis of drinking water health risks from a spill of hydraulic fracturing flowback water. Risk Analysis, 38(4), 724–754. 10.1111/risa.12884 28973831

[gh270015-bib-0147] Rose, L. D. , Akob, D. M. , Tuberty, S. R. , Corsi, S. R. , DeCicco, L. A. , Colby, J. D. , & Martin, D. J. (2019). Use of high‐throughput screening results to prioritize chemicals for potential adverse biological effects within a West Virginia watershed. Science of the Total Environment, 677, 362–372. 10.1016/j.scitotenv.2019.04.180 31059879

[gh270015-bib-0148] Rosenblum, J. , Nelson, A. W. , Ruyle, B. , Schultz, M. K. , Ryan, J. N. , & Linden, K. G. (2017). Temporal characterization of flowback and produced water quality from a hydraulically fractured oil and gas well. The Science of the Total Environment, 596–597, 369–377. 10.1016/j.scitotenv.2017.03.294 28448913

[gh270015-bib-0149] Rosenblum, J. , Thurman, E. M. , Ferrer, I. , Aiken, G. , & Linden, K. G. (2017). Organic chemical characterization and mass balance of a hydraulically fractured well: From fracturing fluid to produced water over 405 days. Environmental Science & Technology, 51(23), 14006–14015. 10.1021/acs.est.7b03362 29132208

[gh270015-bib-0150] Rossi, R. J. , DiGiulio, D. C. , & Shonkoff, S. B. C. (2022). An examination of onshore produced water spills in the state of California: Incident frequency, spatial distribution, and shortcomings in available data. Environmental Science and Pollution Research, 30(7), 18631–18642. 10.1007/s11356-022-23391-0 36215008

[gh270015-bib-0151] Rowan, E. L. , Engle, M. A. , Kraemer, T. F. , Schroeder, K. T. , Hammack, R. W. , & Doughten, M. W. (2015). Geochemical and isotopic evolution of water produced from middle Devonian Marcellus shale gas wells, Appalachian Basin, Pennsylvania. AAPG Bulletin, 99(2), 181–206. Retrieved from https://pubs.geoscienceworld.org/aapgbull/article‐abstract/99/2/181/133456/Geochemical‐and‐isotopic‐evolution‐of‐water?redirectedFrom=fulltext

[gh270015-bib-0152] Sabie, R. P. , Pillsbury, L. , & Xu, P. (2022). Spatiotemporal analysis of produced water demand for fit‐for‐purpose reuse—A permian basin, New Mexico case study. Water, 14(11), 1735. 10.3390/w14111735

[gh270015-bib-0153] Santos, I. C. , Hildenbrand, Z. L. , & Schug, K. A. (2019). A review of analytical methods for characterizing the potential environmental impacts of unconventional oil and gas development. Analytical Chemistry, 91(1), 689–703. 10.1021/acs.analchem.8b04750 30392348

[gh270015-bib-0154] Sappington, E. N. , & Rifai, H. S. (2018). Low‐frequency electromagnetic treatment of oilfield produced water for reuse in agriculture: Effect on water quality, germination, and plant growth. Environmental Science and Pollution Research, 25(34), 34380–34391. 10.1007/s11356-018-3343-x 30302734

[gh270015-bib-0155] Scanlon, B. R. , Reedy, R. C. , & Wolaver, B. D. (2021). Assessing cumulative water impacts from shale oil and gas production: Permian Basin case study. Science of the Total Environment, 811, 152306. 10.1016/j.scitotenv.2021.152306 34906580

[gh270015-bib-0156] Scanlon, B. R. , Reedy, R. C. , Xu, P. , Engle, M. , Nicot, J. P. , Yoxtheimer, D. , et al. (2020). Can we beneficially reuse produced water from oil and gas extraction in the U.S. The Science of the Total Environment, 717, 137085. 10.1016/j.scitotenv.2020.137085 32209263

[gh270015-bib-0157] Schreiber, M. E. , & Cozzarelli, I. M. (2021). Arsenic release to the environment from hydrocarbon production, storage, transportation, use and waste management. Journal of Hazardous Materials, 411, 125013. 10.1016/j.jhazmat.2020.125013 33482508

[gh270015-bib-0158] Sedlacko, E. M. , Heuberger, A. L. , Chaparro, J. M. , Cath, T. Y. , & Higgins, C. P. (2022). Metabolomics reveals primary response of wheat (Triticum aestivum) to irrigation with oilfield produced water. Environmental Research, 212(Pt D), 113547. 10.1016/j.envres.2022.113547 35660401

[gh270015-bib-0159] Shariq, L. (2013). Uncertainties associated with the reuse of treated hydraulic fracturing wastewater for crop irrigation. Environmental Science & Technology, 47(6), 2435–2436. 10.1021/es4002983 23438315

[gh270015-bib-0160] Silva, G. S. , Warren, J. L. , & Deziel, N. C. (2018). Spatial modeling to identify sociodemographic predictors of hydraulic fracturing wastewater injection wells in Ohio census block groups. Environmental Health Perspectives, 126(6). 10.1289/ehp2663 PMC6084846 29957590

[gh270015-bib-0161] Sirivedhin, T. , & Dallbauman, L. (2004). Organic matrix in produced water from the Osage‐Skiatook petroleum environmental research site, Osage county, Oklahoma. Chemosphere, 57(6), 463–469. 10.1016/j.chemosphere.2004.05.034 15350408

[gh270015-bib-0162] Sitterley, K. A. , Linden, K. G. , Ferrer, I. , & Thurman, E. M. (2018). Identification of proprietary amino ethoxylates in hydraulic fracturing wastewater using liquid chromatography/time‐of‐flight mass spectrometry with solid‐phase extraction. Analytical Chemistry, 90(18), 10927–10934. 10.1021/acs.analchem.8b02439 30139247

[gh270015-bib-0163] Skalak, K. J. , Engle, M. A. , Rowan, E. L. , Jolly, G. D. , Conko, K. M. , Benthem, A. J. , & Kraemer, T. F. (2013). Surface disposal of produced waters in western and southwestern Pennsylvania: Potential for accumulation of alkali‐earth elements in sediments. International Journal of Coal Geology, 126, 162–170. 10.1016/j.coal.2013.12.001

[gh270015-bib-0164] Smalling, K. L. , Anderson, C. W. , Honeycutt, R. K. , Cozzarelli, I. M. , Preston, T. , & Hossack, B. R. (2019). Associations between environmental pollutants and larval amphibians in wetlands contaminated by energy‐related brines are potentially mediated by feeding traits. Environmental Pollution, 248, 260–268. 10.1016/j.envpol.2019.02.033 30798027

[gh270015-bib-0165] Smith, K. H. , Mackey, J. E. , Wenzlick, M. , Thomas, B. , & Siefert, N. S. (2024). Critical mineral source potential from oil & gas produced waters in the United States. Science of the Total Environment, 929, 172573. 10.1016/j.scitotenv.2024.172573 38641103

[gh270015-bib-0166] Soriano, M. A. , Deziel, N. C. , & Saiers, J. E. (2022). Regional scale assessment of shallow groundwater vulnerability to contamination from unconventional hydrocarbon extraction. Environmental Science & Technology, 56(17), 12126–12136. 10.1021/acs.est.2c00470 35960643 PMC9454823

[gh270015-bib-0167] Stallworth, A. M. , Chase, E. H. , McDevitt, B. , Marak, K. K. , Freedman, M. A. , Wilson, R. T. , et al. (2021). Efficacy of oil and gas produced water as a dust suppressant. Science of the Total Environment, 799, 149347. 10.1016/j.scitotenv.2021.149347 34426301 PMC8530883

[gh270015-bib-0168] State of New Mexico, & U.S. Environmental Protection Agency . (2018). Oil and natural gas produced water governance in the State of New Mexico ‐ Draft White Paper. New Mexico. Retrieved from https://www.epa.gov/sites/production/files/2018‐11/documents/oil_and_natural_gas_produced_water_governance_in_the_state_of_new_mexico_draft_white_paper_508.pdf

[gh270015-bib-0169] States, S. , Cyprych, G. , Stoner, M. , Wydra, F. , Kuchta, J. , Monnell, J. , & Casson, L. (2013). Marcellus Shale drilling and brominated THMs in Pittsburgh, Pa., drinking water. Journal of the American Water Works Association, 105(8), 53–54. 10.5942/jawwa.2013.105.0107

[gh270015-bib-0170] Struchtemeyer, C. G. , & Elshahed, M. S. (2012). Bacterial communities associated with hydraulic fracturing fluids in thermogenic natural gas wells in North Central Texas, USA. FEMS Microbiology Ecology, 81(1), 13–25. 10.1111/j.1574-6941.2011.01196.x 22066833

[gh270015-bib-0171] Sun, Y. , Wang, D. , Tsang, D. C. W. , Wang, L. , Ok, Y. S. , & Feng, Y. (2019). A critical review of risks, characteristics, and treatment strategies for potentially toxic elements in wastewater from shale gas extraction. Environment International, 125, 452–469. 10.1016/j.envint.2019.02.019 30763832

[gh270015-bib-0172] Tasker, T. L. , Burgos, W. D. , Ajemigbitse, M. A. , Lauer, N. E. , Gusa, A. V. , Kuatbek, M. , et al. (2019). Accuracy of methods for reporting inorganic element concentrations and radioactivity in oil and gas wastewaters from the Appalachian Basin, US based on an inter‐laboratory comparison. Environmental Science‐Processes & Impacts, 21(2), 224–241. 10.1039/c8em00359a 30452047

[gh270015-bib-0173] Tasker, T. L. , Warner, N. R. , & Burgos, W. D. (2020). Geochemical and isotope analysis of produced water from the Utica/point pleasant shale, Appalachian Basin. Environmental Sciences: Processes & Impacts, 22(5), 1224–1232. 10.1039/D0EM00066C 32322852

[gh270015-bib-0174] Texas Commission on Environmental Quality . (2024). Dorchester operating company, LLC, permit No. WQ0005400000. Retrieved from https://www15.tceq.texas.gov/crpub/index.cfm?fuseaction=regent.showSingleRN&re_id=502353292021210

[gh270015-bib-0175] Texas Produced Water Consortium . (2022). Beneficial use of produced water in Texas: Challenges, opportunities and the path forward. Retrieved from https://www.depts.ttu.edu/research/tx‐water‐consortium/downloads/22‐TXPWC‐Report‐Texas‐Legislature.pdf

[gh270015-bib-0176] Texas Produced Water Consortium . (2024). Request for proposals: Analytical support for desalination of Texas oilfield waste produced water. Retrieved from https://www.depts.ttu.edu/research/tx‐water‐consortium/request‐for‐proposals‐rfp.php

[gh270015-bib-0177] Thacker, J. B. , Carlton, D. D. , Hildenbrand, Z. L. , Kadjo, A. F. , & Schug, K. A. (2015). Chemical analysis of wastewater from unconventional drilling operations. Water, 7(4), 1568–1579. 10.3390/w7041568

[gh270015-bib-0178] Thakur, P. , Ward, A. L. , & Schaub, T. M. (2022). Occurrence and behavior of uranium and thorium series radionuclides in the Permian shale hydraulic fracturing wastes. Environmental Science and Pollution Research, 29(28), 43058–43071. 10.1007/s11356-021-18022-z 35091928

[gh270015-bib-0179] Thiel, G. P. , & Lienhard, J. H., V. (2014). Treating produced water from hydraulic fracturing: Composition effects on scale formation and desalination system selection. Desalination, 346, 54–69. 10.1016/j.desal.2014.05.001

[gh270015-bib-0180] Thurman, E. M. , Ferrer, I. , Rosenblum, J. , Linden, K. , & Ryan, J. N. (2017). Identification of polypropylene glycols and polyethylene glycol carboxylates in flowback and produced water from hydraulic fracturing. Journal of Hazardous Materials, 323, 11–17. 10.1016/j.jhazmat.2016.02.041 26947804

[gh270015-bib-0181] Tinker, K. , Gardiner, J. , Lipus, D. , Sarkar, P. , Stuckman, M. , & Gulliver, D. (2020). Geochemistry and microbiology predict environmental niches with conditions favoring potential microbial activity in the Bakken shale. Frontiers in Microbiology, 11. 10.3389/fmicb.2020.01781 PMC7406717 32849400

[gh270015-bib-0182] Tinker, K. , Lipus, D. , Gardiner, J. , Stuckman, M. , & Gulliver, D. (2022). The microbial community and functional potential in the midland basin reveal a community dominated by both thiosulfate and sulfate‐reducing microorganisms. Microbiology Spectrum, 10(4), 000499‐22. 10.1128/spectrum.00049-22 PMC9430316 35695567

[gh270015-bib-0183] Torres, L. , Yadav, O. P. , & Khan, E. (2017). Holistic risk assessment of surface water contamination due to Pb‐210 in oil produced water from the Bakken Shale. Chemosphere, 169, 627–635. 10.1016/j.chemosphere.2016.11.125 27912187

[gh270015-bib-0184] Torres, L. , Yadav, O. P. , & Khan, E. (2018). Risk assessment of human exposure to Ra‐226 in oil produced water from the Bakken Shale. Science of the Total Environment, 626, 867–874. 10.1016/j.scitotenv.2018.01.171 29396348

[gh270015-bib-0185] U.S. Energy Information Administration . (2019). U.S. Shale oil and natural gas maps [Dataset]. EIAMaps: OilandGasExploration, Resources, and Production. Shapefile. https://www.eia.gov/maps/maps.htm

[gh270015-bib-0186] U.S. Environmental Protection Agency . (2020). Summary of input on oil and gas extraction wastewater management practices Under the Clean Water Act (No. EPA‐821‐S19‐001) (p. 38).

[gh270015-bib-0187] U.S. Geological Survey . (2022). 2022 final list of critical minerals: Federal. Register, 87(37), 10381–10382. Retrieved from https://www.federalregister.gov/d/2022‐04027

[gh270015-bib-0188] Van Sice, K. , Cravotta, C. A. , McDevitt, B. , Tasker, T. L. , Landis, J. D. , Puhr, J. , & Warner, N. R. (2018). Radium attenuation and mobilization in stream sediments following oil and gas wastewater disposal in western Pennsylvania. Applied Geochemistry, 98, 393–403. 10.1016/j.apgeochem.2018.10.011

[gh270015-bib-0189] Varonka, M. S. , Gallegos, T. J. , Bates, A. L. , Doolan, C. , & Orem, W. H. (2020). Organic compounds in produced waters from the Bakken formation and three Forks formation in the Williston Basin, north Dakota. Heliyon, 6(3), e03590. 10.1016/j.heliyon.2020.e03590 32195404 PMC7076043

[gh270015-bib-0190] Veil, J. (2015). U.S. Produced water volumes and management practices in 2012. e Ground Water Protection Council. Retrieved from https://www.gwpc.org/wp‐content/uploads/2022/12/Produced_Water_Report_2014_GWPC_0_1.pdf

[gh270015-bib-0191] Veil, J. (2020). U.S. Produced water volumes and management practices in 2017. Ground Water Research and Education Foundation. https://www.gwpc.org/wp‐content/uploads/2020/02/pw_report_2017___final.pdf

[gh270015-bib-0192] Vikram, A. , Lipus, D. , & Bibby, K. (2016). Metatranscriptome analysis of active microbial communities in produced water samples from the Marcellus Shale. Microbial Ecology, 72(3), 571–581. 10.1007/s00248-016-0811-z 27457653

[gh270015-bib-0193] Wang, H. (2021). Shale oil production and groundwater: What can we learn from produced water data? PLoS One, 16(4), e0250791. 10.1371/journal.pone.0250791 33930038 PMC8087075

[gh270015-bib-0194] Wang, H. , Lu, L. , Chen, X. , Bian, Y. , & Ren, Z. J. (2019). Geochemical and microbial characterizations of flowback and produced water in three shale oil and gas plays in the central and western United States. Water Research, 164, 114942. 10.1016/j.watres.2019.114942 31401327

[gh270015-bib-0195] Wang, N. , Kunz, J. L. , Cleveland, D. , Steevens, J. A. , & Cozzarelli, I. M. (2019). Biological effects of elevated major ions in surface water contaminated by a produced water from oil production. Archives of Environmental Contamination and Toxicology, 76(4), 670–677. 10.1007/s00244-019-00610-3 30850858

[gh270015-bib-0196] Warner, N. R. , Christie, C. A. , Jackson, R. B. , & Vengosh, A. (2013). Impacts of shale gas wastewater disposal on water quality in western Pennsylvania. Environmental Science & Technology, 47(20), 11849–11857. 10.1021/es402165b 24087919

[gh270015-bib-0197] Warner, N. R. , Darrah, T. H. , Jackson, R. B. , Millot, R. , Kloppmann, W. , & Vengosh, A. (2014). New tracers identify hydraulic fracturing fluids and accidental releases from oil and gas operations. Environmental Science & Technology, 48(21), 12552–12560. 10.1021/es5032135 25327769

[gh270015-bib-0198] Warner, N. R. , Kresse, T. M. , Hays, P. D. , Down, A. , Karr, J. D. , Jackson, R. B. , & Vengosh, A. (2013). Geochemical and isotopic variations in shallow groundwater in areas of the Fayetteville Shale development, north‐central Arkansas. Applied Geochemistry, 35, 207–220. 10.1016/j.apgeochem.2013.04.013

[gh270015-bib-0199] Weaver, J. , Xu, J. , & Mravik, S. (2015). Scenario analysis of the impact on drinking water intakes from bromide in the discharge of treated oil and gas wastewater. Journal of Environmental Engineering, 142(1), 04015050. 10.1061/(ASCE)EE.1943-7870.0000968

[gh270015-bib-0200] Welch, S. A. , Sheets, J. M. , Daly, R. A. , Hanson, A. , Sharma, S. , Darrah, T. , et al. (2021). Comparative geochemistry of flowback chemistry from the Utica/Point Pleasant and Marcellus formations. Chemical Geology, 564, 120041. 10.1016/j.chemgeo.2020.120041

[gh270015-bib-0201] Wilson, J. M. , & VanBriesen, J. M. (2012). Oil and gas produced water management and surface drinking water sources in Pennsylvania. Environmental Practice, 14(04), 288–300. 10.1017/S1466046612000427

[gh270015-bib-0208] Willis, M. D. , Carozza, S. E. , & Hystad, P. (2022). Congenital anomalies associated with oil and gas development and resource extraction: a population‐based retrospective cohort study in Texas. Journal of Exposure Science & Environmental Epidemiology. 10.1038/s41370-022-00505-x PMC9852077 36460921

[gh270015-bib-0202] Wright, M. T. , McMahon, P. B. , Landon, M. K. , & Kulongoski, J. T. (2019). Groundwater quality of a public supply aquifer in proximity to oil development, Fruitvale oil field, Bakersfield, California. Applied Geochemistry, 106, 82–95. 10.1016/j.apgeochem.2019.05.003

[gh270015-bib-0203] Yao, Y. , Chen, T. , Shen, S. S. , Niu, Y. , DesMarais, T. L. , Linn, R. , et al. (2015). Malignant human cell transformation of Marcellus Shale gas drilling flow back water. Toxicology and Applied Pharmacology, 288(1), 121–130. 10.1016/j.taap.2015.07.011 26210350 PMC4698968

[gh270015-bib-0204] Zhang, T. , Hammack, R. W. , & Vidic, R. D. (2015). Fate of radium in Marcellus shale flowback water impoundments and assessment of associated health risks. Environmental Science & Technology, 49(15), 9347–9354. 10.1021/acs.est.5b01393 26154523

[gh270015-bib-0205] Ziemkiewicz, P. F. (2013). Characterization of liquid waste streams from shale gas development. AGH Drilling, Oil, Gas, 30(1), 297. 10.7494/drill.2013.30.1.297

[gh270015-bib-0206] Ziemkiewicz, P. F. , & He, T. Y. (2015). Evolution of water chemistry during Marcellus shale gas development: A case study in West Virginia. Chemosphere, 134(0), 224–231. 10.1016/j.chemosphere.2015.04.040 25957035

[gh270015-bib-0207] Ziemkiewicz, P. F. , Quaranta, J. D. , Darnell, A. , & Wise, R. (2014). Exposure pathways related to shale gas development and procedures for reducing environmental and public risk. Journal of Natural Gas Science and Engineering, 16, 77–84. 10.1016/j.jngse.2013.11.003

