# Supplementary material for: Pathways for Potential Exposure to Onshore Oil and Gas Wastewater: What We Need to Know to Protect Human Health
Source: Geohealth. 2025 Apr 3;9(4):e2024GH001263. doi: 10.1029/2024GH001263 (PMC11966568; doi:10.1029/2024GH001263)
Supplement: Supplementary file 1 — Supporting Information S1 [file GH2-9-e2024GH001263-s001.docx]

*GeoHealth*

Supporting Information for

**Pathways for potential exposure to onshore oil and gas wastewater: What we need to know to protect human health**

Ayusha Ariana^1^, Isabelle Cozzarelli^2^, Cloelle Danforth^1^, Bonnie McDevitt^2^, Anna Rosofsky^1^, Donna Vorhees^1^

^1^Health Effects Institute Energy, Boston, MA, USA

^2^Geology, Energy & Minerals Science Center, U.S. Geological Survey, Reston, VA, USA

**Contents of this file**

Tables S1 to S2

**Additional Supporting Information (Files uploaded separately)**

Table S1. Summary table of produced water studies referenced in this review organized by location, type, **exposure medium**, data used for analysis, and the type of oil and gas production (n = 121).

Table S2. Summary table of **produced water composition** studies referenced in this review organized by location, type, aim of study, data used for analysis, and the type of oil and gas production (n = 115).

**Introduction**

Included in this Supplementary Information are all the produced water studies referenced in the review, organized by exposure (Table S1) and chemical composition (Table S2). These summary tables of include the types of information available in these resources that is potentially relevant to researchers and decision-makers

| **Study** | **State** | **Shale or Basin** | **Study Type** | **Exposure Medium** | **Data** | **Unconventional (UOGD) vs. Conventional Oil & Gas Development (COGD)** |
| --- | --- | --- | --- | --- | --- | --- |
| Abraham et al. 2023 | TX | N/A | Water modeling | Drinking water | Samples of produced water and surface water used for drinking water | N/A |
| Abualfaraj et al. 2018 | PA, WV, NY | Marcellus | Human health risk assessment | Drinking water | Samples from a freshwater reservoir into which flowback water was known to be spilled | UOGD |
| Akob et al. 2016 | WV | Appalachian, Marcellus | Water monitoring | Surface water, sediments, microbiology | Samples of surface water near Class II Underground Injection Control (UIC II) wells disposal facility | Both |
| Allen et al. 2013 | National | Appalachian, Gulf Coast, Midcontinent, Rocky Mountain | Air monitoring | Air | Methane measurements from different well equipment at several development and production stages | Both |
| Anders et al. 2022 | CA | Orcutt Oil Field | Water monitoring | Groundwater | Samples of groundwater and historical oil well data | COGD |
| Balise et al. 2019a | N/A | N/A | Toxicology | Hydraulic fracturing chemicals | Samples of 23 commonly used hydraulic fracturing chemicals | UOGD |
| Balise et al. 2019b | N/A | N/A | Toxicology | Hydraulic fracturing chemicals | Samples of 23 commonly used hydraulic fracturing chemicals | UOGD |
| Bain et al. 2021 | WV, OH, PA | Appalachian | Exposure assessment using water modeling | Road treatment | Samples of produced water treated for use as a deicer and dust suppressor on roads in a residential area | COGD |
| Bean et al. 2018 | TX | Wolfcamp, Permian | Water modeling | Air | Airborne emissions measured from flowback stored on the well site | UOGD |
| Bonetti et al. 2021 | National | N/A | Water monitoring | Surface water | Samples of surface water and geocoded data on well density | UOGD |
| Boulé et al. 2018 | N/A | N/A | Toxicology | Hydraulic fracturing chemicals | Samples of 23 commonly used hydraulic fracturing chemicals | UOGD |
| Burgos et al. 2017 | PA | Marcellus | Water monitoring, Soil monitoring | Groundwater, sediment | Samples of groundwater and sediment potentially contaminated by insufficiently treated produced water | UOGD |
| Cantlay et al. 2019 | ND, PA | Bakken, Marcellus | Water monitoring | Surface water, Groundwater | Samples of surface water, impoundment water, flowback, produced water, and mine drainage | Both |
| Cantlay et al. 2020 | ND, PA | Bakken, Marcellus | Water monitoring | Surface water, Groundwater | Samples of surface water, impoundment water, flowback, produced water, and mine drainage | Both |
| Casey et al. 2022 | MD, PA, OH, WV, VA | Marcellus | Water monitoring | Surface water | Samples of surface water and geocoded data on both COGD and UOGD well density | Both |
| Chilkoor et al. 2018 | ND | Bakken | Water monitoring, Water modeling | Agricultural soil, Surface water | Samples of flowback | UOGD |
| Cozzarelli et al. 2021 | ND | Williston | Water monitoring, sediment monitoring, biological effects | Surface water, sediments | Samples of surface water and sediments known to be contaminated by a wastewater spill | UOGD |
| Cozzarelli et al. 2017 | ND | Williston | Water monitoring, sediment monitoring, biological effects | Surface water, sediments | Samples of surface water and sediments known to be contaminated by a wastewater spill | UOGD |
| DiGiulio and Jackson 2016 | WY | Wind River Formation | Water monitoring | Drinking water | Samples of domestic drinking water wells and produced water | UOGD |
| DiGiulio et al. 2021 | CA | Tulare | Water monitoring | Groundwater | Samples of groundwater in the vicinity of produced water ponds | COGD |
| Drollette et al. 2015 | PA | Marcellus | Water monitoring | Groundwater | Samples from private groundwater residential wells hypothesized to be contaminated by produced water and flowback stored in containment pits | UOGD |
| Farag et al. 2022 | ND | Williston | Water monitoring, sediment monitoring, biological effects | Surface water, sediments, fish | Samples of surface water and sediment known to be contaminated by a produced water spill | UOGD |
| Farnan et al. 2023 | PA | N/A | Water monitoring | Surface water (that may be used as drinking water) and sediment | Samples of produced water treated for use as road treatment | N/A |
| Ferrar et al. 2013 | PA | Marcellus | Water monitoring | Treated produced water discharged to surface water | Samples of effluent discharged from three wastewater treatment plants that processed OG wastewater | UOGD |
| Geeza et al. 2018b | PA | Marcellus | Sediment monitoring, Toxicology | Surface water and sediment, mussels | Samples of surface sediment and mussel shell in surface water bodies receiving National Pollutant Discharge Elimination System (NPDES) discharges | UOGD |
| Graber et al. 2017 | ND | Bakken, Three Forks | Air monitoring | Air | Samples of dust from passive dust collectors positioned at various distances from a road applied with produced water | N/A |
| Gross et al. 2013 | CO | Denver-Julesburg | Water monitoring | Groundwater | Samples of groundwater potentially contaminated from surface spills of produced water from active well sites | N/A |
| Harkness et al. 2017 | WV | Marcellus | Water monitoring | Groundwater, surface water | Samples of groundwater and surface water collected before, during, and after hydraulic fracturing in a shale gas development area. | UOGD |
| Hladik et al. 2014 | CO, MD, PA, VA | N/A | Water monitoring | Treated produced water discharged to surface water | Samples of surface water collected around wastewater treatment and disposal facilities | COGD |
| Huang et al. 2018 | PA | Marcellus | Water modeling | Surface water | Samples of surface water injected with wastewater | UOGD |
| Johnson et al. 2022 | WV | N/A | Air monitoring | Air | Emissions measured from evaporated produce water in onsite storage tanks | N/A |
| Johnson et al. 2015 | NY, PA | Marcellus | Water monitoring | Groundwater, surface water | Samples of groundwater and surface water in an oil and gas-dense region | N/A |
| Johnston et al. 2016 | TX | Eagle Ford | Socio-  economic | N/A | Locations of wastewater disposal wells and socioeconomic data | N/A |
| Kanno and McCray 2021 | CO | Denver-Julesburg | Water modeling | Groundwater | Colorado Energy and Carbon Management Commission (ECMC) data on reported spills of produced water | UOGD |
| Kassotis et al. 2020 | CO | N/A | Water monitoring | Groundwater, surface water | Measurements of endocrine bioactivities and UOGD geochemical tracers of wastewater in groundwater and surface water samples | UOGD |
| Kassotis et al. 2016a | N/A | N/A | Toxicology | Hydraulic fracturing chemicals | Samples of 23 commonly used hydraulic fracturing chemicals | UOGD |
| Kassotis et al. 2018a | CO, WV | Marcellus, Piceance | Toxicology | Hydraulic fracturing chemicals, Surface water, produced water | Samples of 23 commonly used hydraulic fracturing chemicals, surface water contaminated by produced water or near produced water treatment and disposal facilities, produced water from storage infrastructure | UOGD |
| Kassotis et al. 2016b | WV | Fayetteville | Toxicology | Surface water | Samples of surface water near UIC II disposal facility | UOGD |
| Kassotis et al. 2015 | CO | N/A | Toxicology | Wastewater | Three samples of wastewater taken from a leaking pipeline and storage tanks | UOGD |
| Kassotis et al. 2014 | CO | Colorado River Drainage, Piceance | Toxicology, Water monitoring | Hydraulic fracturing chemicals, Surface water, Groundwater | Samples of surface water and groundwater in a drilling-dense region | N/A |
| Kassotis et al. 2018b | WY | Marcellus | Toxicology, Water monitoring | Hydraulic fracturing chemicals, Groundwater | Samples of hydraulic fracturing chemicals and groundwater at various distances from oil and gas development | Both |
| Kharaka et al. 2005 | OK | N/A | Water monitoring | Groundwater | Samples of produced water and groundwater potentially contaminated by nearby produced water | N/A |
| Kharaka et al. 2007 | OK | N/A | Water monitoring | Groundwater | Samples of groundwater potentially contaminated by nearby produced water | N/A |
| Kingsbury et al. 2023 | PA, WV | N/A | Water monitoring, Water modeling | Surface water | Samples of surface water in an oil and gas-dense region | N/A |
| Kohl et al. 2014 | PA | Marcellus | Water monitoring | Groundwater, drinking water | Samples of groundwater (used as drinking water) collected before and after hydraulic fracturing nearby | UOGD |
| Kondash et al. 2020 | CA | N/A | Water monitoring, Soil monitoring | Plant uptake | Samples of groundwater, produced water treated for irrigation, and soil irrigated by produced water | COGD |
| Landis et al. 2016 | PA | Marcellus | Water monitoring | Surface water | Samples of surface water potentially contaminated by insufficiently treated wastewater | Both |
| Lauer et al. 2016 | ND | Bakken, Williston | Water monitoring, Soil monitoring | Surface water, soil | Samples of surface water, produced water, and soil | UOGD |
| Lauer et al. 2018 | PA | Marcellus | Soil monitoring | Sediment in surface water near wastewater disposal sites | Samples of surface water sediment | COGD |
| LeDoux et al. 2016 | KY | Central Appalachian | Water monitoring | Groundwater | Samples of groundwater near UOGD | UOGD |
| Li et al. 2016 | CO | Denver-Julesburg | Water monitoring | Groundwater | 672 samples of groundwater from the ECMC GIS database, of which some are suspected of contamination due to thermogenic methane, and 514 samples of produced water from Colorado State University | UOGD |
| Llewellyn et al. 2015 | PA | Marcellus | Water monitoring | Drinking water | Samples of aquifer groundwater hypothesized to be contaminated with flowback water | UOGD |
| Lyman et al. 2018 | UT, WY | Uintah, Upper Green River | Air monitoring | Air | Emission measurements from produced water impoundments taken over a 3-year period | N/A |
| Ma et al. 2019 | PA, WV | Marcellus | Exposure assessment using water modeling | Groundwater, soil, air | Estimated volume of produced water and potential chemical pathways of constituents | UOGD |
| Ma et al. 2022 | PA, WV | Marcellus | Human health risk assessment | Groundwater, soil, air | Return water from several horizontal wells | UOGD |
| Maloney et al. 2017 | CO, ND, NM, PA | N/A | Water monitoring | Groundwater, soil, air | Samples of return water from several horizontal wells | UOGD |
| Mansfield et al. 2018 | UT, WY | Uinta, Upper Green River | Air monitoring | Air | Emission measurements from produced water impoundments taken over a 3-year period | N/A |
| McDevitt et al. 2019 | WY | N/A | Water monitoring, sediment monitoring | Surface water, sediment | Samples of surface water and sediment receiving NPDES-permitted discharges | N/A |
| McDevitt et al. 2020b | WY | Undisclosed | Water monitoring | Surface water | Samples of treated produced water discharged to surface water intended for irrigation | Both |
| McDevitt et al. 2021b | WY | Undisclosed | Water monitoring, sediment monitoring | Surface water, sediments, plant uptake | Samples from 3 NPDES discharge facilities and 5 wetlands | N/A |
| McLaughlin et al. 2020b | WY | Undisclosed | Water monitoring | N/A | Samples of surface water collected downstream of discharge location of produced water treated for irrigation | N/A |
| McLaughlin et al. 2020a | WY | Undisclosed | Exposure assessment using water modeling | Surface water | Samples of surface water from streams receiving NPDES-permitted discharges of treated produced water for beneficial reuse | N/A |
| McLaughlin et al. 2021 | WY | Undisclosed | Water monitoring | Surface water | Samples of produced water undergoing passive treatment in constructed wetlands | N/A |
| McLimans et al. 2022 | PA | Marcellus | Toxicology | Surface water | Samples of liver tissue from native Brook trout (*Salvelinus fontinalis)* | N/A |
| McMahon et al. 2019b | CA | San Joaquin Valley | Water monitoring | Groundwater | Samples of groundwater proximate to COGD | COGD |
| McMahon et al. 2019a | NY, PA | Marcellus | Water monitoring | Groundwater | Samples of groundwater in 50 domestic wells in upland areas of the Marcellus region | UOGD |
| McMahon et al. 2017 p. 201 | TX | Eagle Ford, Fayetteville Shale, Haynesville Shale | Water monitoring | Groundwater | Samples of groundwater from 116 wells in an UOGD-dense region | UOGD |
| Michaels et al. 2022 | WV | Marcellus | Water modeling and monitoring, Soil modeling | Surface water bodies with UOGD in their watersheds, soil | Samples of stream water sediment in an UOGD-dense region | UOGD |
| Miller et al. 2020 | CO | Denver-Julesburg | Water monitoring, Soil monitoring | Agricultural soils and produced water-surface water blend irrigated crops | Samples of soils and wheat irrigated with a blend of produced water and surface water | N/A |
| Mumford et al. 2020 | PA | Marcellus | Water monitoring | Surface water | Samples of surface water potentially contaminated by produced water | UOGD |
| Murray-Gulde et al. 2003 | N/A | N/A | Water monitoring, Toxicology | Produced water, broadleaf cattail, giant bulrush (planted in wetlands) | Samples of produced water and specified plants before and after passing through wetland treatment system | N/A |
| Nagel et al. 2020 | N/A | N/A | Toxicology, Review | Hydraulic fracturing chemicals | Review of studies based on samples of 23 commonly used hydraulic fracturing chemicals, produced water, and potentially contaminated surface and groundwater | UOGD |
| Oetjen et al. 2018a p. 201 | CO | Denver-Julesburg | Soil modeling | Soil, food (crop) | Simulated produced water spill onto agricultural soil | UOGD |
| Orem et al. 2017 | PA | Marcellus | Water monitoring, sediment monitoring | Surface water | Samples of surface water near UIC II disposal facility | UOGD |
| Osborn and McIntosh 2010 | KT, NY, OH, PA | Appalachia | Water monitoring | Groundwater, Drinking water | 61 samples of natural gas, 24 samples of co-produced formation water, and thirteen samples of groundwater used as drinking water | UOGD |
| Pancras et al. 2015 | PA | Marcellus | Water monitoring | Surface water | Samples of surface water upstream and downstream of Centralized Waste Treatment facilities, outfalls from Centralized Waste Treatments, coal-fired electrical generating stations, and other industrial sources | Both |
| Parker et al. 2014 | AR, OH, PA, CA | Fayetteville, Marcellus | Water monitoring | Surface water, Drinking water | Samples of surface water, municipal wastewater effluent, flowback, and produced water | UOGD |
| Patnode et al. 2015 | PA | Marcellus | Toxicology, Water monitoring | Surface water | Samples of surface water collected by the Environmental Protection Agency and Pennsylvania Department of Environmental Protection upstream and downstream of wastewater treatment facilities | N/A |
| Pelak and Sharma 2014 | PA | Marcellus | Water monitoring | Surface water | Samples of surface water from 50 streams in a UOGD-dense region | UOGD |
| Peterman et al. 2012 | MT | Bakken | Soil monitoring, Water monitoring | Groundwater, surface water, soil | Samples of soil from wetlands, groundwater, and surface water and produced water collected from monitoring wells, wetlands, and an oil/brine separation tank | N/A |
| Pfister et al. 2017 | TX | Permian | Water monitoring | Groundwater | Samples of groundwater and produced water from an enhanced oil recovery site | COGD |
| Phan et al. 2018 | TX, WV | Marcellus, Permian | Water monitoring | Groundwater, produced water | Samples of groundwater and produced water from two enhanced oil recovery sites | N/A |
| Piotrowski et al. 2020 | OH | Utica | Toxicology | Produced water | One sample of produced water and samples of mussel shell and tissue | UOGD |
| Preston et al. 2014 | MT | Williston | Water monitoring and modeling | Groundwater and surface water potentially contaminated by produced water | Samples of groundwater and surface water in an energy development-dense region | N/A |
| Preston et al. 2019 | MT | Williston | Water monitoring | Groundwater and surface water potentially contaminate by produced water | Samples of groundwater and surface water in an energy development-dense region | N/A |
| Ramirez 2002 | WY | Undisclosed | Water monitoring | Surface water | Samples of surface water from wetland sites receiving NPDES-permitted discharges of treated produced water for beneficial reuse | N/A |
| Redmon et al. 2021 | CA | N/A | Human health risk assessment | Food (crop) | Samples of produced water treated for irrigation | COGD |
| Reilly et al. 2015 | PA | Marcellus | Water monitoring | Drinking water | Samples of flowback water and drinking water from 21 residential water wells | N/A |
| Rich and Crosby 2013 | TX | Barnett | Soil monitoring, Water monitoring | Soil, groundwater | Samples of soil and produced water from “reserve pits” (impoundments/ponds) | UOGD |
| Rish and Pfau 2018 | PA, WV | Marcellus | Human health risk assessment | Drinking water | Samples of flowback water from 19 shale gas wells | N/A |
| Rossi et al. 2022 | CA | N/A | Water monitoring | N/A | Produced water spill records | Both |
| Rossi et al. 2023 | CA | San Joaquin Valley | Water monitoring | Surface water | State database of samples from produced water storage ponds | N/A |
| Sappington and Rifai 2018 | TX | Eagle Ford | Water monitoring | Plant uptake (via irrigation) | Samples of treated and untreated produced water used to irrigate crops | N/A |
| Scanlon et al. 2021 | TX | Permian | Water monitoring | Groundwater, surface water | Samples of groundwater and surface water contaminated by produced water spills | UOGD |
| Sedlacko et al. 2022 | CO | Denver-Julesburg | Water monitoring, Soil monitoring | Treated produced water irrigated crops | Measurements of development outcomes of crops irrigated by treated produced water | UOGD |
| Shrestha et al. 2017 | MT, ND, Canada | Bakken | Water modeling | Groundwater | Samples of produced water | UOGD |
| Shonkoff et al. 2016 | CA | San Joaquin Valley | Water monitoring, Toxicology | Groundwater, plant uptake (via irrigation) | Samples of oil field produced water treated for irrigation, livestock watering, and groundwater recharge | COGD |
| Shores and Laituri 2018 | UT, WY | Uinta, Upper Green River | Air monitoring | Air | Emission measurements from produced water impoundments taken over a 3-year period | N/A |
| Shores et al. 2017 | CO | Denver-Julesburg | Water modeling | Groundwater | Produced water spill records | UOGD |
| Silva et al. 2018 | OH | N/A | Socio-economic | N/A | Locations of UIC II injection wells | N/A |
| Skalak et al. 2013 | PA | Marcellus | Soil monitoring and modeling | Sediment potentially contaminated by treated produced water from wastewater treatment plants | Samples of sediment surrounding wastewater treatment plants | Both |
| Smalling et al. 2019 | ND, MT | Williston | Sediment monitoring, Toxicology, Water monitoring | Sediment | Samples of sediment, surface water, and various species of amphibians from wetlands | N/A |
| Soriano et al. 2022 | PA, OH, WV | Appalachian | Water modeling | Groundwater | No samples taken; Modeling groundwater flow and contaminant transport | UOGD |
| Stallworth et al. 2021 | National | N/A | Air modeling | Air | Samples of produced water treated to suppress dust on roads, simulated PM10 and total dissolved solids levels in response to treatment | N/A |
| Stanton et al. 2023 | CA | Montebello Oil Field | Water monitoring | Groundwater | Samples of groundwater near an oil field | COGD |
| States et al. 2013 | PA | Marcellus | Water monitoring | Drinking water, surface water | Samples of surface water and drinking water | N/A |
| Tasker et al. 2018 | National | N/A | Water monitoring, Toxicology | Surface water and sediment | Samples of produced water used for road treatment | Both |
| Tisherman et al. 2023 | CA | San Joaquin Valley | Water monitoring | Groundwater | Samples of produced water from produced water storage ponds and samples of groundwater in their vicinity | N/A |
| Torres et al. 2018 | ND | Bakken | Exposure assessment using water modeling | Food (fish, crop) consumption, Drinking water | Simulated Ra-226 levels in produced water based on correlations with Sr, Ba, Ca- ions | UOGD |
| Torres et al. 2017 p. 201 | ND | Bakken Shale | Exposure assessment using water modeling | Surface water, drinking water | Modeled pathways of lead isotope Pb-210 reaching drinking water through produced water spills into surface water using data from Lauer et al. (2016) | UOGD |
| Van Sice et al. 2018 | PA | Marcellus | Soil monitoring | Sediment in surface water | Samples of sediment cores in surface water bodies around wastewater treatment plants | Both |
| Vengosh et al. 2013 | AR, PA, OH, TX, WV | Utica, Marcellus, Fayettesville, Haynesville | Water monitoring | Groundwater | Review of studies looking at water quality impacts | UOGD |
| Wang 2021 | NM | Permian | Water monitoring | Groundwater | Samples of produced water from active wells | Both |
| Warner et al. 2013a | PA | Marcellus | Water monitoring, Soil monitoring | Surface water, sediment | Samples of effluent from wastewater treatment plants to surface water and sediments | Both |
| Warner et al. 2013b | AR | Fayetteville Shale | Water monitoring | Groundwater, drinking water | Samples of groundwater in shallow aquifers overlying producing shale formations | N/A |
| Weaver et al. 2015 | PA | Marcellus | Water modeling | Drinking water | Samples of surface water around commercial wastewater treatment plant | Both |
| Webb et al. 2014 | N/A | N/A | Toxicology, Review | Hydraulic fracturing chemicals, produced water, surface and drinking water water potentially contaminated by produced water | Review of studies providing evidence on how adult and early life exposure to UOGD chemicals can result in adverse reproductive health and developmental effects in humans from UOGD operations | UOGD |
| Wilson and Van Briesen 2013 | PA, WV | Marcellus | Water monitoring | Surface water, drinking water | Samples of surface water taken over a 3-year period | Both |
| Wilson and VanBriesen 2012 | PA | Marcellus | Water modeling | Surface water, drinking water | No samples taken; data from the Pennsylvania Department of Environmental Protection | Both |
| Wright et al. 2019 | CA | Fruitvale Oil Field | Water monitoring | Drinking water | Samples of groundwater and produced water | N/A |
| Zhang et al. 2015 | PA | Marcellus | Exposure assessment using water modeling | Flowback | Samples of flowback water over a 2.5-year period | N/A |

Table S1. Summary table of produced water studies referenced in this review organized by location, type, exposure medium, data used for analysis, and the type of oil and gas production (n = 121).

| **Study** | **State** | **Shale or Basin** | **Type of water** | **Data** | **Unconventional (UOGD) vs. Conventional Oil & Gas Development (COGD)** |
| --- | --- | --- | --- | --- | --- |
| Abualfaraj et al. 2014 | PA, NY | Marcellus | Flowback, produced water | Database of 35,000 entries of flowback and produced water sampling data from the Environmental Protection Agency, Gas Technology Institute, Pennsylvania Department of Environmental Protection, Bureau of Oil and Gas Management, and the New York Department of Environmental Conservation | N/A |
| Acharya et al. 2020 | N/A | N/A | Flowback, produced water | Review of various studies,  biological treatment | UOGD |
| Aghababaei et al. 2021 | N/A | Utica, Marcellus | Produced water | 42 samples of input media and produced water from four wells | N/A |
| Akob et al. 2015 | PA | Devonian, Marcellus, Burket | Produced water | 13 samples of produced water from gas-liquid separator tanks | N/A |
| Akyon et al. 2018 | N/A | Bakken, Utica | Produced water | Seven samples of produced water from separators from oil and gas wells in both shales, biological treatment | UOGD |
| Al-Ghouti et al. 2019b | National | N/A | Produced water | Review of various studies | N/A |
| Barbot et al. 2013 | PA | Marcellus | Produced water | 160 samples of flowback and produced water | UOGD |
| Barnaby et al. 2004 | NM | Permian | Formation water | Samples of formation water collected from 71 wells | N/A |
| Bern et al. 2021 | NY, ND, TX, CO | National, Marcellus, Bakken, Barnett, Niobara | Produced water | United States Geological Survey (USGS) Produced Water Geochemical Database, samples of produced water from specified shales and states | Both |
| Blauch et al. 2009 | PA | Marcellus | Flowback | More than 100 flowback analyses collected over eighteen months | UOGD |
| Blondes et al. 2020 | OH, PA | Utica | Produced water | Chemical analyses of produced water data from the USGS Produced Water Database | UOGD |
| Booker et al. 2017 | OH | Utica | Produced water | Samples of produced water collected from one well for over 200 days, microbial characteristics | UOGD |
| Capo et al. 2014 | PA | Marcellus | Produced water | Samples of flowback and produced water from five wells | UOGD |
| Chapman et al. 2012 | PA | Marcellus | Produced water | Samples of produced water collected from wellheads and impoundments | UOGD |
| Chaudhary et al. 2019 | NM | Permian | Produced water | USGS Produced Water Database and data from the Mew Mexico Water and Infrastructure Data System | UOGD |
| Chen et al. 2023b | CO, NM, TX, WY | Anadarko, Permian, Raton, San Juan, Uinta, Williston | Produced water | Review of various studies | N/A |
| Chittick and Srebotnjak 2017 | CA | N/A | Produced water | Data compiled from individual well reports submitted by operators | UOGD |
| Cluff et al. 2014 | PA | Marcellus | Flowback, Produced water | Three samples of injected fluid from holding tanks, 25 samples of flowback from the wellhead, and six samples of produced water from wellheads and gas-fluid separators | UOGD |
| Conrad et al. 2020 | National | Marcellus | Produced water | USGS Produced Water Database | UOGD |
| Crosby et al. 2018 | PA | Marcellus | Produced water | Samples of produced water from two UOGD and two COGD wells | Both |
| Daly et al. 2016 | OH, PA | Marcellus, Utica | Produced water, injected fluid | Samples of produced water and injected fluids collected from wellheads and oil/gas separators | UOGD |
| Danforth et al. 2020 | National | N/A | Produced water | Review of various studies | Both |
| Dresel and Rose 2010 | PA | Marcellus | Formation water | 40 samples of formation water from various parts of Western Pennsylvania | N/A |
| Engle et al. 2020 | TX | Eagle Ford | Produced water, formation water | 39 samples of produced water | UOGD |
| Engle et al. 2016 | NM, TX | Permian | Produced water | 39 samples of produced water from UOGD and previously published COGD data | Both |
| Engle and Rowan 2014 | PA, WV | Marcellus | Produced water | Samples of injected water and produced water from 19 UOGD wells | UOGD |
| Fan et al. 2018 | MI, PA | Antrim, Marcellus, Utica-Collingwood | Flowback, produced water | Samples of flowback taken from downstream of the wellhead and upstream of the storage tank in the Utica-Collingwood formation and seventeen samples of produced water from the Antrim shale | UOGD |
| Ferrer and Thurman 2015 | CO | Denver-Julesburg | Flowback, produced water | Samples of flowback and produced water collected from different locations in Weld County | UOGD |
| Gallegos et al. 2021 | ND, MT | Williston | Produced water | Samples of flowback and produced water | UOGD |
| Gieg et al. 2010 | AK | N/A | Produced water | Two samples from two oil fields | COGD |
| Goldberg and Griffith 2017 | TX | Barnett | Flowback, produced water | Samples of flowback, produced water, and surface water, surface water, and groundwater from various locations overlying and within the shale | UOGD |
| Haluszczak et al. 2013 | PA | Marcellus | Flowback | 22 samples of flowback collected by the Pennsylvania Department of Environmental Protection and Bureau of Oil and Gas Management, samples of flowback from two wells, and data from 40 COGD wells sampled in 1985 | Both |
| Harkness et al. 2015 | PA, WV | Fayetteville, Marcellus | Produced water | 44 samples of produced water from COGD, 25 samples from UOGD in the Marcellus, 6 samples from UOGD in the Fayetteville Formation, and samples of effluent of treated oil and gas wastewater discharged to surface water in PA | Both |
| Hayes 2009 | PA, WV | Marcellus | Flowback, produced water | Data on produced water characterization from 17 member companies of the Marcellus Shale Coalition | UOGD |
| He et al. 2016b | PA | Marcellus | Flowback | Samples of flowback from sites in northeastern PA | UOGD |
| He et al. 2018 | PA | Marcellus | Flowback, produced water | Modeling analysis based on datasets collected from Kondash et al. (2016) and Rowan et al. (2015) | UOGD |
| Hildenbrand et al. 2018 | TX | Eagle Ford | Flowback, produced water, and related wastes | Fourteen samples before, during, and after treatment from various sites in the shale formation, biological treatment | UOGD |
| Hoelzer et al. 2016 | AR | Fayetteville | Produced water | Six samples of produced water from the Arkansas Oil and Gas Commission | UOGD |
| Hu et al. 2022 | NM | Permian | Produced water | Three samples of produced water | N/A |
| Jiang et al. 2021 | NM | Permian | Produced water | USGS Produced Water Database and data on produced water quantity from the New Mexico Oil Conservation Division | Both |
| Jiang et al. 2022 | NM, TX | Permian | Produced water, surface water | Samples of produced water collected from the wellhead, separator, storage tanks or ponds, and the back end of the disposal tank battery system | UOGD |
| Jubb et al. 2020 | National | Eagle Ford, Marcellus, Permian, Utica/Point Pleasant, Williston | Produced water | Fifteen samples from the listed reservoirs collected from various USGS field campaigns | Unspecified |
| Khan et al. 2016 | TX | Permian | Produced water | Samples of produced water from eight wells in the Wolfcamp Formation | UOGD |
| Kharaka et al. 2019 | CO | Denver-Julesburg, Raton, Trinidad, Greely | Produced water | USGS Produced Water Database and data from the Colorado Oil and Gas Conservation Commission | Both |
| Kim et al. 2016 | CO | Denver- Julesburg | Flowback, produced water | Samples of flowback water and produced water collected from two wells | UOGD |
| Kim et al. 2019 | CO | Denver-Julesburg | Produced water | Samples of produced water from wells hydraulically fractured with different types of fracturing fluid | UOGD |
| Kim et al. 2020 | CO | N/A | Produced water treated for industry reuse, hydraulic fracturing fluid | Samples of recycled water from a produced water treatment facility, hydraulic fracturing fluid, and tap water and fresh water used as source water for hydraulic fracturing. | UOGD |
| Kim et al. 2016 | CO | N/A | Flowback, produced water | Samples of flowback and produced water collected from two wells | UOGD |
| Lester et al. 2015 | CO | Denver-Julesburg | Flowback | One sample of flowback taken by an operator | UOGD |
| Liang et al. 2016 | TX | Barnett | Produced water | Samples of produced water collected from a shale gas production field | UOGD |
| Liden et al. 2022 | TX | Permian, Eagle Ford | Produced water | 24 samples of produced water from different well equipment on various well pads | UOGD |
| Lipus et al. 2017 | PA | Marcellus | Produced water | 42 samples of produced water | UOGD |
| Lipus et al. 2018 | ND | Bakken, Three Forks | Produced water | Samples of produced water collected from separators and storage tanks on seventeen different well sites | UOGD |
| Lipus et al. 2019 | PA | Marcellus | Produced water | Two samples of produced water from impoundments in Washington County and a third from a hauling truck | UOGD |
| Luek et al. 2017 | CO, ND, OH, PA, WV | Appalachian, Bakken, Denver-Julesburg, Marcellus, Utica | Flowback, produced water, natural gas compression and liquid fractionation wastewater | Sixteen samples of various wastewaters | UOGD |
| Luek et al. 2018 | WV | Marcellus | Produced water, Flowback, Hydraulic fracturing fluid | Time series samples of produced water, flowback water, and hydraulic fracturing fluid from two adjacent wells | UOGD |
| Luek and Gonsior 2017 | PA, CO, WV, TX, AR, OH, NM, IN, KY (National) | Marcellus, DJ, Burkett, Fayetteville, Permian, Utica, Barnett, New Albany | Hydraulic fracturing fluid, flowback, produced water | Review of various studies | UOGD |
| Maguire-Boyle and Barron 2014 | PA, NM, TX | Marcellus, Eagle Ford, Barnett | Flowback, produced water | Samples of produced water from the three shales | UOGD |
| Macpherson et al. 2014 | PA | Marcellus | Produced water | Samples of produced water collected at wellhead, separator, and storage tanks | UOGD |
| Macpherson 2015 | AL, OH, PA, TX | Appalachia, Gulf Coast Sedimentary Basin | Produced water | Samples of produced water collected by University of Pittsburgh, DOE, and USGS | UOGD |
| McDevitt et al. 2020a | PA | Marcellus | Flowback, acid mine drainage discharge | Flowback from two wells and four acid mine drainage discharges | UOGD |
| McDevitt et al. 2022 | National | Bakken, Denver-Julesburg, Eagle Ford, Marcellus, Permian, Williston | Produced water | Produced water samples collected from 18 wells | UOGD |
| McMahon et al. 2018 | CA | San Joaquin Valley | Formation water, Produced water | Samples of water and gas in 22 oil wells, produced water disposed of in three injection wells, and produced water from two surface disposal ponds in four oil fields | N/A |
| Murali Mohan et al. 2013a | PA | Marcellus | Injected fluid, hydraulic fracturing fluid, produced water, synthetic oil-based drilling mud | Samples of various wastewaters collected from a single well over time | UOGD |
| Murali Mohan et al. 2013b | PA | Marcellus | Flowback | Samples of flowback taken from three impoundments both untreated and treated to various extents | UOGD |
| Nell and Helbling 2019 | WV | Marcellus | Flowback, produced water | Fourteen samples of flowback and produced water, FracFocus for information on hydraulic fracturing chemicals | UOGD |
| Nelson et al. 2015 | PA | Marcellus | Produced water | One sample of produced water from northeastern PA | UOGD |
| Nicot et al. 2018 | TX | Eagle Ford | Produced water | Samples of produced water from fifteen wells the investigators sampled directly, as well as samples from four different companies |  |
| Oetjen and Thomas 2016 | PA, WV | Marcellus | Flowback | Flowback data from the Shale Network | Both |
| Oetjen et al. 2018b | CO | DJ Basin | Flowback, produced water | Flowback and produced water samples from one well over time | UOGD |
| Ogbuji et al. 2022 | TX | Permian | Produced water | USGS produced water datasets and geochemical data sets from 45 oil and gas operations | UOGD |
| Orem et al. 2014 | PA, IN, WY, MT, ND | Marcellus, New Albany, Williston Basin | Produced water, formation water | Review of various studies | UOGD |
| Ouyang et al. 2019 | PA | Marcellus | Flowback, produced water, solid wastes | One sample of untreated produced water from a waste treatment facility, which was separated into liquid and solid to obtain a raw waste solid sample, a sample of waste treatment solids precipitated from mixing two different hydraulic fracturing wastewaters and three discharges from abandoned mine drainage samples, and six additional samples of waste treatment solids precipitated from mixing flowback from two different Marcellus wells with three discharges from abandoned mine drainage at different proportions | UOGD |
| Phan et al. 2016 | PA | Marcellus, Appalachia | Produced water | Samples of produced water from 3 COGD wells and 5 UOGD wells | Both |
| Phan et al. 2015 | PA | Marcellus | Produced water, formation water | 43 samples of produced water from 10 wells, and 15 samples of formation water | UOGD |
| Piotrowski et al. 2018a | PA | Marcellus | Produced water, Flowback | Samples of flowback collected from four wells | UOGD |
| Piotrowski et al. 2018b | PA | Marcellus | Flowback, hydraulic fracturing fluid | Samples of hydraulic fracturing fluids and its source water, and four samples of flowback from a single well | UOGD |
| Quillinan et al. 2018 | National | N/A | Produced water | 224 samples of produced water and 101 rock samples | Both |
| Regnery et al. 2016 | CO | Denver-Julesburg | Produced water | 27 samples of produced water analyzed over eight weeks and treated by osmosis-reverse osmosis hybrid treatment system | UOGD |
| Rosenblum et al. 2017a | CO | Denver-Julesberg | Flowback, produced water | Nine samples of flowback and produced water from a storage tank of one well taken over time | UOGD |
| Rosenblum et al. 2017c | CO | Denver- Julesburg | Flowback, produced water, hydraulic fracturing fluid | Nine samples of produced water and flowback, and samples of the injected fluids from one well measured over 405 days | UOGD |
| Rowan et al. 2011 | PA | Marcellus | Produced water | Three time-series and 13 grab samples of produced water | UOGD |
| Rowan et al. 2015 | PA, NY | Marcellus | Produced water | Samples of produced water from various sources and state databases | Both |
| Roychaudhuri et al. 2019 | PA | Marcellus | Flowback | Three samples of flowback, of which one is treated, and the others are treated with different technologies to differing extents | UOGD |
| Schreiber and Cozzarelli 2021 | National | Marcellus, Eagle Ford, Bakken, Antrim, New Albany | Produced water | Review of various studies | Both |
| Sharma et al. 2020 | National | Trenton, Edwards, Wilcox, Lance, Marcellus, Bakken, Mesaverde | Produced water | USGS Produced Water Geochemical Database v2.3 | Both |
| Shih et al. 2015 | PA | Marcellus | Produced water, flowback, drilling wastes | Compiled dataset of 160 samples of produced water, flowback, and drilling wastes from state-required wastewater generator reports filed in 2009-2011 | UOGD |
| Sirivedhin and Dallbauman 2004 | OK | N/A | Produced water, groundwater | Three samples including from an oil/coalbed methane well, two oil wells, and one sample of groundwater from a well on site | N/A |
| Sitterley et al. 2018 | CO, OK, ND, TX, WY | N/A | Flowback, produced water | 20 samples of flowback and produced water collected from various shale deposits in the United States | UOGD |
| Stewart et al. 2015 | NY | Marcellus | Produced water | Nine samples of dry-drill cuttings were used to understand leachates and how they influence produced water composition | UOGD |
| Stringfellow and Camarillo 2019 | CA | N/A | Produced water | Data from mandatory reporting of characterization of produced water and injected fluids from well simulation | UOGD |
| Strong et al. 2014 | ND, PA | Bakken, Marcellus | Produced water, flowback, hydraulic fracturing fluid | One sample of produced water collected from a well hydraulically fractured 18 months prior in PA, a sample of hydraulic fracturing fluid from PA, two samples of flowback from ND | UOGD |
| Struchtemeyer and Elshahed 2012 | TX | Barnett | Injected fluid, flowback | One sample of “frac” pond water stored for use in hydraulic fracturing, one sample of frac water treated with biocides, one sample of frac water supplied with hydraulic fracturing chemicals, and one sample of flowback from the separator | UOGD |
| Sun et al. 2019 | National | Bakken, Barnett, Eagle Ford, Marcellus, Montney, Duverney | Flowback, produced water | Review of various studies | UOGD |
| Tasker et al. 2020 | PA, WV, OH | Utica/Point Pleasant, Marcellus | Produced water | Samples of produced water from 26 wells | UOGD |
| Taylor et al. 2018 | PA | Marcellus | Flowback, produced water | Eight samples of flowback and produced water from onsite tanks and impoundments | UOGD |
| Thacker et al. 2015 | TX | N/A | Wastewater (unspecified) | Three samples of wastewater, one directly from effluent from a well, one from a disposal well, and another from a waste pit | UOGD |
| Thakur et al. 2022 | NM, TX | Permian | Flowback, produced water, waste proppant sand | Seven samples of flowback and produced water and three samples of waste proppant sand from storage tanks | UOGD |
| Thiel and Lienhard V 2014 | NY, PA, TX | Marcellus, Permian | Produced water | Three samples of produced water from the Permian and five samples from the Marcellus | UOGD |
| Tinker et al. 2020 | ND | Bakken | Produced water | Samples from 14 wells in the Bakken, microbial analysis | UOGD |
| Tinker et al. 2022 | TX, NM | Permian, Midland | Produced water | Samples from 10 wells in the Permian and Midland basins, microbial analysis | UOGD |
| Thurman et al. 2014 | CO, LA,  NV, PA,  TX | Denver-Julesburg, Barnett, unspecified for LA, NV, and PA | Flowback, produced water | Six samples of flowback and six samples of produced water from various locations collected by industry and government personnel | UOGD |
| Thurman et al. 2017 | CO | Denver-Julesburg | Flowback, produced water | Samples of flowback and produced water from four different wells collected at different points of the production phase | Both |
| Varonka et al. 2020 | ND | Bakken Formation, Three Forks Formation, Williston Basin | Produced water | 12 samples of produced water from 12 wells | UOGD |
| Vikram et al. 2016 | PA | Marcellus | Produced water | Three samples of produced water, two from separate impoundments and one from a truck transporting produced water directly from the wellhead | UOGD |
| Wang et al. 2019a | CO, ND, TX | Denver-Julesburg, Bakken, Barnett | Flowback, produced water | Samples of flowback and produced water collected from several different wells | UOGD |
| Warner et al. 2014 | AR, PA, WV | Bakken, Marcellus | Produced water, flowback, hydraulic fracturing fluid, groundwater, wastewater treatment facility effluent | 39 samples of produced water from both COGD and UOGD, one sample of hydraulic fracturing fluid, 15 samples of flowback from two wells in the Marcellus and six wells in the Fayetteville Formation, samples of shallow groundwater from a salt spring overlying the Marcellus, samples of effluent of treated wastewater discharged to surface water, one sample of surface water collected at an accidental spill site | Both |
| Welch et al. 2021 | OH, WV | Utica/Point Pleasant, Marcellus | Flowback, produced water | Samples of flowback and produced water from two wells in the Utica/ Point Pleasant shale and one in the Marcellus | UOGD |
| Welch et al. 2022 | OH | Utica Shale/Point Pleasant | Flowback, produced water | Samples of flowback and produced water from five wells on two well pads | UOGD |
| Xiao 2021 | ND | Bakken | Produced water | Samples of produced water from oil fields | COGD |
| Yao et al. 2015 | PA | Marcellus | Flowback | Samples of flowback from five wells | UOGD |
| Zielinski and Budahn 2007 | OK | N/A | Produced water | Two samples of produced water collected from a storage tank at a USGS research site | COGD |
| Ziemkiewicz 2013 | PA, WV | Marcellus | Hydraulic fracturing fluid, flowback, produced water, and drilling muds and cuttings | Review of various studies | UOGD |
| Ziemkiewicz et al. 2014 | WV | Marcellus | Flowback, drilling muds, hydraulic fracturing fluids | Samples of flowback, drilling muds, and hydraulic fracturing fluids analyzed for exposure pathways | UOGD |
| Ziemkiewicz and He 2015 | WV | Marcellus | Hydraulic fracturing fluid, flowback, produced water | Samples of flowback, hydraulic fracturing fluid, and produced water from four wells taken over time | UOGD |

Table S2. Summary table of produced water composition studies referenced in this review organized by location, type, aim of study, data used for analysis, and the type of oil and gas production (n = 115).
